# Marine influence on airborne bacterial community composition and predicted functional potential in coastal zones: a case study from Ostend, Belgium

**DOI:** 10.1038/s41598-026-54664-0

**Published:** 2026-05-22

**Authors:** Yunmeng Li, Zixia Liu, Pascal I. Hablützel, Wannes Van Beeck, Colin R. Janssen, Sarah Lebeer, Jana Asselman, Maarten De Rijcke

**Affiliations:** 1 Flanders Marine Institute (VLIZ), InnovOcean Campus, Jacobsenstraat 1, 8400 Ostend, Belgium; 2 Blue Growth Research Lab, Ghent University, Wetenschapspark 1, 8400 Ostend, Belgium; 3 Laboratory of Applied Microbiology and Biotechnology, Department of Bioscience Engineering, University of Antwerp, Groenenborgerlaan 171, 2020 Antwerp, Belgium; 4 Department of Biology, Vrije Universiteit Brussel, Pleinlaan 2, 1050 Brussels, Belgium

**Keywords:** Airborne bacteria, Coastal aerosols, Predicted functional potential, Potential habitat affiliations, Air mass back trajectories, Nanopore sequencing, Ecology, Ecology, Environmental sciences, Microbiology, Ocean sciences

## Abstract

Airborne bacteria in coastal zones and the marine influence on them remain poorly understood. Here, we investigated airborne bacterial communities at a coastal site in Ostend, Belgium, during late spring and summer using Nanopore full-length 16S rRNA gene sequencing. By comparing glass and quartz fiber filters across multiple sampling durations, we found that quartz filters with 4–12 h sampling provided sufficient DNA while avoiding longer sampling durations that may introduce bias. These samples were characterized by air mass trajectories, environmental conditions, and comparison with bacteria detected in local seawater. Air mass origin and temperature were associated with bacterial community composition. Samples associated with oceanic air masses, particularly under conditions favorable for sea spray aerosol generation, tended to show greater overlap with bacteria detected in local seawater. *Proteobacteria*, *Firmicutes*, *Bacteroidota*, and *Actinobacteriota* predominated across samples. Marine-influenced aerosols tended to show higher relative abundance of *Gammaproteobacteria* and higher predicted representation of pathways related to protein export and diverse metabolic processes, whereas less marine-influenced samples showed greater representation of chemotaxis- and xenobiotic-associated metabolism pathways. Potential habitat affiliations suggested stronger ocean-related signatures in marine-influenced aerosols and broader terrestrial affiliations in less marine-influenced samples. These findings highlight marine-terrestrial interactions in shaping coastal airborne bacterial communities.

**Supplementary Information:**

The online version contains supplementary material available at 10.1038/s41598-026-54664-0.

## Introduction

Over the past decade, airborne bacteria in outdoor environments have attracted growing attention for their impacts on public health and agriculture, their roles in atmospheric chemistry (e.g., degradation of organic compounds), and their influence on climate as cloud condensation nuclei^1,2^. Although extensive research has been conducted in inland areas, coastal zones remain relatively underexplored^2^. This is notable given that a large portion of the global population lives within 5 km of coastlines^3,4^, and millions visit beaches annually for recreational activities^5^. Understanding airborne bacterial communities in coastal zones is therefore important for assessing their ecological roles and potential health relevance.

Airborne bacterial communities are influenced by various factors, such as emission sources, air mass trajectories, and environmental conditions^1,2,6^. Research by Tignat-Perrier et al. (2019) indicated that these communities primarily result from nearby sources, with diluted contributions from distant sources^7^. Located at the ocean-land interface, coastal zones are influenced by both seawater and diverse terrestrial environments such as soils, vegetation, and urban infrastructure. When wind speeds exceed 4 m/s, a condition favorable for the generation of sea spray aerosols (SSAs)^8^, millions of microbes per m^2^ of ocean surface may be released into the atmosphere daily^9^. These microbes, carried by ocean winds, may contribute to airborne bacterial communities in coastal zones. Mesocosm studies have revealed that bacterial transfer from seawater to air via SSAs is selective and taxon-specific^10–12^. In addition, recent studies comparing bacteria in marine aerosols with those in the underlying sea surface microlayer and seawater^13–17^, as well as airborne bacteria associated with marine versus terrestrial air masses^18^, have provided further insight into aerosolization patterns and the potential contribution of marine bacteria to atmospheric processes and air quality. However, how marine and terrestrial influences jointly shape airborne bacterial communities in coastal zones remains poorly understood.

The advent of high-throughput sequencing (HTS) techniques, including next-generation sequencing (NGS, since 2006) and third-generation sequencing (TGS, since 2011), has advanced our knowledge of airborne bacterial communities^2,19^. As summarized in Table S1, HTS-based studies of airborne bacterial communities in open seas and coastal zones remain limited, despite a gradual increase over the past seven years. This is primarily due to the low biomass of airborne bacteria in these regions, making it difficult to obtain sufficient material for sequencing^20,21^. Airborne bacterial concentrations over oceans typically range from 10^3^ to 10^5^ cells/m^3^ air, 10–1000 times lower than inland levels (10^5^ to 10^6^ cells/m^3^ air)^22–26^. Extended sampling times are often used, but this can lead to cell rupture and nucleic acid degradation, as well as environmental changes, compromising the representativeness of the samples^27,28^. To date, the classical air filtration method (i.e., drawing air through a filter) remains widely used, with sampling durations ranging from several hours to weeks, and filter types varying in materials, porosity, and pore size (Table S1). Studies comparing filter types for collecting specific airborne bacterial strains or chemicals (e.g., phycotoxins) in laboratory chamber systems have shown different collection efficiencies, assessed by particle counts or chemical concentrations^29–31^. Filter comparisons for microbial communities under environmental conditions are, however, scarce^32^. This is particularly relevant in low-biomass coastal aerosols, where sampling strategy may affect DNA yield and sample representativeness, thereby influencing subsequent community analyses.

Few coastal aerosol studies have examined whether marine influence is reflected in the taxonomic composition and predicted functional potential of airborne bacterial communities. Here, we investigated airborne bacterial communities in a coastal zone using Nanopore full-length 16S rRNA gene sequencing. Aerosol samples were collected on glass and quartz fiber filters for various durations on the rooftop of a coastal site located approximately 300 m from the coastline. We first evaluated filter types and sampling durations by comparing DNA yield, bacterial diversity, and community composition, and then selected samples for further community analysis based on these results. These samples were further characterized in relation to air mass sources, environmental conditions, and their overlap with bacteria detected in local seawater. We hypothesized that (1) samples influenced by oceanic air masses, particularly under conditions favorable for SSA generation, would show greater overlap with bacteria detected in local seawater, and (2) such samples would differ in community composition and predicted functional potential from less marine-influenced samples.

## Materials and methods

### Sample collection

Aerosol samples were collected from May 25 to September 15, 2021, on the rooftop of the Marine Station Ostend (MSO) in Ostend, Belgium (Figure S1A). The sampling setup included an AirCube HE high-volume air sampler (AMS Analytica; hereafter ‘AirCube’), positioned indoors near the roof (Figure S1C). Air was drawn through a tube with an inner diameter of 9 mm that split into two equal-length branches via a Y-joint. Each branch was connected to a 47-mm in-line stainless steel filter holder (Pall Corporation). The two holders were mounted side by side on a 1-m-high iron pole fixed to the rooftop (Figure S1B), enabling simultaneous sampling. To improve aerosol collection efficiency, the frontal support screens of the holders were removed as recommended by Van Acker et al.^31^. Two filter types were tested: glass fiber (EPM2000) and quartz fiber (QM-A) (Table S2). Both filter types are commonly used for aerosol sampling (Table S1) and are known for high retention efficiency (99.95% for ≥ 0.3 µm particles) and mechanical robustness against clogging and breakage during prolonged sampling (Table S2). Despite similar specifications, previous studies have reported differences in their performance for collecting aerosolized phycotoxins and sodium (a proxy for SSA content^33^)^31^, suggesting that their performance may also differ for airborne bacterial sampling. These filters were therefore compared in this study. To minimize instrumental bias, the two holders were used alternately, and filter-tube connections were switched between sampling events. The AirCube flow rate was set to 50 L/min, while the actual airflow through each filter was measured using a rotameter (SKC, 320-440) before and after sampling, confirming consistent flow rates across filters (~ 22 L/min each). Sampling was conducted in several batches with durations ranging from 1 to 72 h. Each duration was performed separately to account for the high temporal variability in aerosol particles and airborne bacterial counts reported in previous studies^34,35^, which may affect the biomass available for sequencing. The inlets of the holders were oriented towards the prevailing wind direction and adjusted during daytime if major shifts occurred. Each sample filter was paired with a control filter that underwent the same manipulations but was not connected to the air sampler. These control filters were used to assess potential contamination associated with filter handling, transport, and processing. Specifically, both sample and control filters were placed in separate holders, sealed in individual plastic bags (DaklaPack, 324), and transported to the sampling site. During sampling, the control filters remained sealed. After sampling, the holders containing the sample filters were resealed in their plastic bags and transported to the laboratory at MSO together with the control filters for processing. Filters were carefully removed from the holders using precleaned tweezers, placed in plastic petri dishes (diameter 47 mm, Sigma-Aldrich), and sealed with parafilm (Sigma-Aldrich). All operations were conducted in a laminar flow hood (Thermo Scientific). The filters were then stored at − 86 °C until DNA extraction. All contact materials were either single-use or thoroughly cleaned with 1% (v/v) HNO_3_ (Chem-Lab; 65% purity), 70% (v/v) ethanol (Chem-Lab), and Milli-Q water (Merck-Millipore) to reduce salt residues and bacterial contamination, and then air-dried in the laminar flow hood before use.

The first batch (24 samples in total), comprising EPM2000 and QM-A filters across 12 distinct durations (1, 2, 3, 4, 6, 8, 12, 24, 36, 48, 58.7, and 72 h), was used to compare filters and identify suitable sampling durations. Subsequent batches used only the selected filter and durations (12 samples in total) for DNA extraction and sequencing. Detailed sample information, including sampling date, time, duration, and total air volume, is provided in Table S3.

### Sample characterization

To assess whether aerosol samples influenced by oceanic air masses showed greater overlap with bacteria detected in local seawater, we analyzed air mass trajectories, environmental conditions during each sampling period, and the relative abundance of aerosol operational taxonomic units (OTUs) also detected in local seawater.

Air mass trajectories were calculated at 11 m above ground level, corresponding to the approximate sampling inlet height (the 10-m rooftop height plus the 1-m iron pole), using the MeteoInfo GIS software with the TrajStat plugin and meteorological data from the Global Data Assimilation System^36^. We used 72 h back trajectories to characterize regional air mass transport while limiting the uncertainty associated with longer trajectory durations. Trajectories corresponding to specific sampling durations were also analyzed as described by Tignat-Perrier et al. (2019), who emphasized the influence of proximate sources^7^. Each aerosol sample was then categorized according to (i) the geographic type of its air mass trajectories (Ocean, Land, Ocean_Land, and Land_Ocean) and (ii) the proportion of time spent over oceanic regions, defined in 10% intervals to quantify oceanic exposure. A detailed description of the classification is provided in Table S4.

Environmental parameters, including ocean variables (e.g., seawater temperature, wave height) and meteorological factors (e.g., air temperature, wind speed), were recorded at 30 min and 10 min intervals, respectively, by local monitoring stations: the ‘Buoy’ (~ 1.2 km from MSO) and the ‘Weather station’ (~ 200 m from MSO) (Figure S1A). The data were obtained from the public database ‘*Meetnet Vlaamse Banken’* (https://meetnetvlaamsebanken.be/), and the average environmental conditions for each sample are summarized in Table S5.

For each aerosol sample collected in this study (spring and summer 2021; Table S3), overlap with bacteria detected in local seawater was calculated as the summed relative abundance of aerosol OTUs that shared the same OTU identity with OTUs detected in local seawater samples collected during the corresponding seasons in a previous study (9 samples in spring and 8 in summer). The seawater samples were collected weekly from March 2018 to March 2019 at knee depth in the surf zone of a recreational sandy beach (~ 400 m from MSO; Figure S1A) and were sequenced and analyzed by Li et al.^37^ using a protocol nearly identical to that applied in the present study. Because the seawater and aerosol samples were collected in different years, this comparison should be interpreted as overlap with a seasonal local reference community rather than as direct evidence of source origin. Previous studies have shown recurrent temporal patterns in marine bacterial communities across years^38,39^, which provides some support for the use of this reference dataset, although interannual variability remains a limitation. Only seawater samples collected during the same seasons as the aerosols were included in this comparison, because bacterial communities in surf zone seawater exhibit strong seasonal variation driven by environmental factors such as seawater temperature^37^. In this study, seawater temperatures during summer aerosol sampling (18.62–20.21 °C, *n* = 12) were higher than during spring sampling (12.37–17.73 °C, *n* = 12) (Table S5).

### Bacterial community analysis

Total DNA was extracted from three-quarters of each sample and control filter using the Qiagen DNeasy Blood & Tissue kit with some modifications as described in Li et al.^37^. DNA yield was quantified using the Qubit dsDNA HS assay kit with a Qubit 3.0 fluorometer (Thermo Fisher Scientific). The full-length 16S rRNA gene was amplified and sequencing libraries were prepared following the protocol described in Li et al.^37^, using the SQK-16S024 16S barcoding kit (barcodes 1–24) and the ligation sequencing kit (SQK-LSK109). Due to the low DNA yield from aerosol samples, 40 PCR cycles were used to obtain sufficient PCR product for library preparation. For samples that still yielded insufficient product after 40 cycles, the original DNA extract was amplified in triplicate, and the resulting PCR products were purified and pooled for library preparation. Sequencing was performed on FLO-MIN106D R9 flow cells using a portable MinION Mk1C device. Sequencing was initiated through MinKNOW (version 21.11.7) using the fast base-calling model and a *Q* score of ≥ 7 to enable near-real-time monitoring of sequence output. After sequencing, the raw FAST5 files were re-base-called using the high-accuracy base-calling model, followed by demultiplexing and barcode trimming to obtain FASTQ data for subsequent processing and analysis. Subsequent sequence processing followed the workflow detailed in Li et al.^37^ and included FASTQ filtering with NanoFilt^40^ (version 2.8.0) (*Q* score ≥ 8; read length 1400–1700 bp), error correction and consensus sequence generation using ASHURE^41^ with 10 iterations, chimera removal using VSEARCH^42^ (version 2.22.1), and taxonomic assignment using BLASTn^43^ (version 2.13.0) against the SILVA v138.1 database, retaining matches with ≥ 90% identity and an alignment length ≥ 1200 bp. Because consensus sequences were generated on a sample-by-sample basis, OTUs were not defined using a fixed sequence-identity clustering threshold across all samples. Instead, for cross-sample comparison, each non-chimeric consensus sequence was assigned the SILVA subject ID of its best BLASTn hit, and sequences sharing the same subject ID were treated as the same OTU. OTUs classified as chloroplast, mitochondria, or lacking phylum-level annotation were discarded. OTUs with fewer than 5 reads were also removed to reduce noise. Table S6 summarizes DNA yield, PCR replicates, band visibility, purified PCR product quantities, sequencing batches, barcode assignments, and read counts before and after filtering.

Rarefaction curves based on observed OTUs were generated to evaluate sequencing sufficiency, and OTU counts were subsequently rarefied to the minimum sequencing depth for alpha diversity estimation using richness (i.e., observed OTUs) and the Shannon index. Beta diversity was assessed using Bray–Curtis dissimilarities calculated from relative abundance data, visualized by principal coordinate analysis (PCoA), and tested using permutational multivariate analysis of variance (PERMANOVA). Although Bray–Curtis dissimilarity calculated from relative abundance data is widely used in microbiome research, it does not fully account for the compositional structure of the data. The beta-diversity results should therefore be interpreted with this limitation in mind. Unless otherwise stated, permutation-based analyses were performed with 999 permutations. Homogeneity of multivariate dispersion among groups was assessed using betadisper followed by permutation tests prior to interpreting PERMANOVA results. Relationships between bacterial community structure and environmental factors were explored using canonical correspondence analysis (CCA) of OTU count data. Prior to CCA, detrended correspondence analysis (DCA) was performed to determine the appropriate ordination method. The longest gradient length of the first DCA axis was 11.22 (> 4.0), supporting the use of a unimodal ordination method (CCA) rather than a linear-based method such as redundancy analysis (RDA). Because the number of environmental variables was large relative to sample size, stepwise forward selection was applied to identify significant variables and derive a reduced model. The significance of the final CCA model and selected variables was then assessed by permutation tests. The predicted functional potential of bacterial communities was inferred using PICRUSt2, which employs phylogenetic placement of 16S rRNA sequences to predict gene families and associated metabolic pathways, with functional annotations derived from the KEGG database (https://www.kegg.jp/kegg/kegg1.html)^44–46^. During sequence placement, input sequences with an alignment proportion below 0.8 to the reference alignment were excluded by the place_seqs.py step. Prediction reliability was then assessed using the nearest-sequenced taxon index (NSTI) provided by PICRUSt2, and only sequences with NSTI values ≤ 0.15 were retained for downstream functional analyses. Potential habitat affiliations were predicted using ProkAtlas, which conducts BLASTn searches against its environmentally categorized 16S rRNA gene sequences to identify likely habitats based on significant matches^47^. Default parameters (97% sequence similarity and 150 bp alignment length) were used. Detected habitats were classified into ocean-related, freshwater-related, soil- and plant-related, human-related, wastewater-related, industrial-related, extreme, and other environments, following the classification scheme of Yokoyama and Kikuchi^48^ with minor modifications (Table S7).

### Additional statistical analyses

Differences in total DNA yield, bacterial richness, and Shannon diversity between the two filter types were evaluated using paired Wilcoxon signed-rank tests based on matched samples collected at the same sampling durations. Differences in the relative abundance of the 10 most abundant phyla were evaluated in the same way, and *p*-values for multiple phylum-level comparisons were adjusted using the Benjamini–Hochberg method. All statistical analyses were performed in R (version 4.2.2), and *p* < 0.05 was considered statistically significant.

## Results and discussion

### Selection of the QM-A filter and 4–12 h sampling duration for subsequent analyses

To compare the efficacy of glass fiber (EPM2000) and quartz fiber (QM-A) filters in capturing airborne bacterial communities and to identify suitable sampling durations, 12 pairs of filters and corresponding blanks were analyzed using Nanopore full-length 16S rRNA gene sequencing across durations of 1, 2, 3, 4, 6, 8, 12, 24, 36, 48, 58.7, and 72 h. Total DNA and PCR-amplified 16S rRNA gene yields in all blanks were below the detection limit, indicating negligible background contamination (Table S6).

**Fig. 1:**
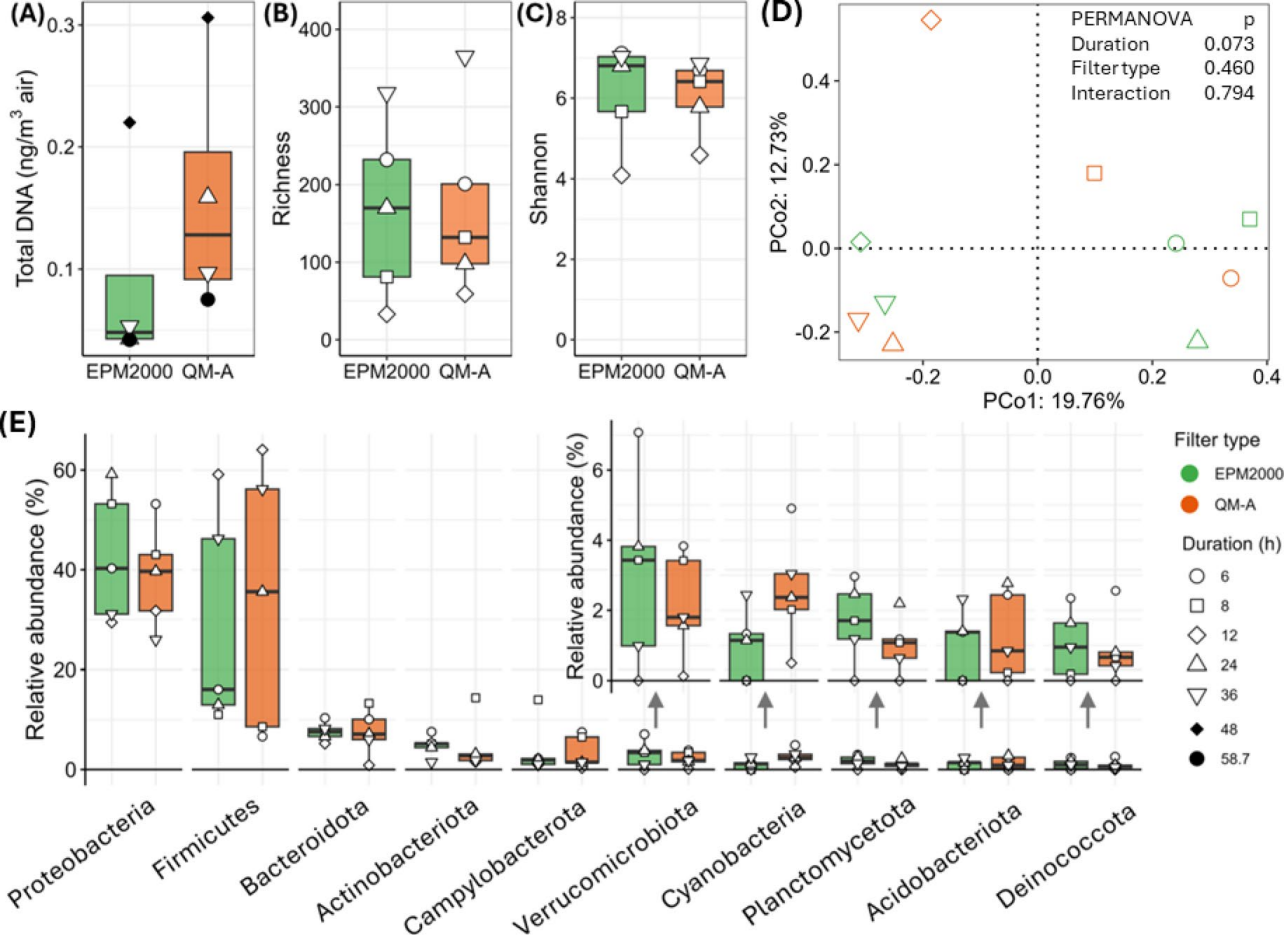
Comparison of EPM2000 and QM-A filters for airborne bacterial community analysis. (**A**) Total DNA; (**B**) Richness (i.e., observed OTUs); (**C**) Shannon diversity index; (**D**) PCoA plot based on Bray–Curtis dissimilarities calculated from relative abundance data; (**E**) Relative abundance of the 10 most abundant phyla. In the boxplots, the center line indicates the median, the box limits represent the 25th and 75th percentiles, and the whiskers extend to the most extreme values within 1.5 × the interquartile range.

Among the 12 filter pairs, samples collected for 24, 36, 48, and 58.7 h yielded detectable DNA (Table S6), with QM-A showing numerically higher DNA yields than EPM2000 in all paired samples (paired Wilcoxon signed-rank test, *p* = 0.125; Fig. 1A). Samples collected with both filters for 6, 8, 12, 24, and 36 h reached sufficient sequencing depth (≥ 3,972 reads per sample; Figure S2, Table S6), as their rarefaction curves plateaued, indicating adequate diversity coverage. Bacterial richness and Shannon diversity did not differ significantly between filters (paired Wilcoxon signed-rank test, both *p* = 1; Fig. 1B,C). Bacterial community composition did not differ significantly between filter types based on PERMANOVA (*R*^*2*^ = 0.105, *p* = 0.460; Fig. 1D), and multivariate dispersion did not differ significantly between the two filter types (permutation test, *p* = 0.220). Likewise, no significant differences in the relative abundances of the 10 most abundant phyla were detected between filter types after Benjamini–Hochberg correction (Fig. 1E).

Given the numerically higher DNA yield in QM-A samples and the lack of clear differences in bacterial diversity and community composition between the two filter types, the QM-A filter was selected. To balance sequencing sufficiency with the aim of minimizing prolonged sampling, which may compromise sample representativeness, a sampling duration of 4–12 h was selected for subsequent analyses.

### Aerosol samples associated with oceanic air masses tended to show higher overlap with bacteria detected in local seawater

Following the selection of the QM-A filter and a 4–12 h sampling duration, 12 samples from the remaining batches meeting these criteria were sequenced together with their blanks (Table S6). These samples, together with three samples from the first batch (0526_2020_Q6, 0527_1601_Q8, 0528_0800_Q12), were included in the subsequent analyses. All samples yielded sufficient sequencing data, and background contamination was negligible (Figure S2, Table S6).

**Fig. 2:**
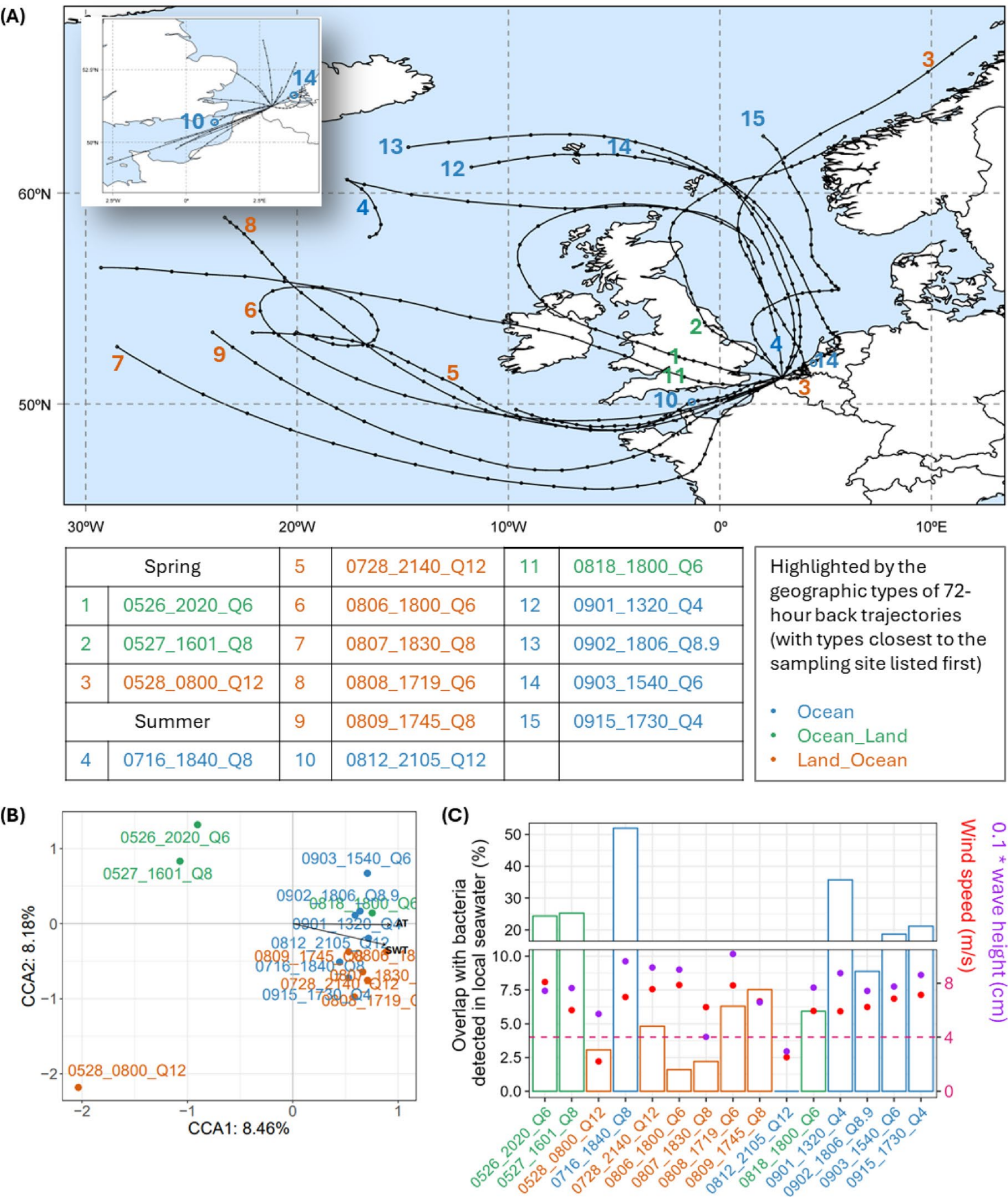
(**A**) 72 h back trajectories (dots indicating 3 h intervals) and sampling-duration-specific back trajectories (inset map; dots indicating 1 h intervals) for aerosol samples. (**B**) Canonical correspondence analysis (CCA) of bacterial community composition in aerosol samples, with samples colored by geographic type based on 72 h back trajectories. Forward selection identified seawater temperature (SWT) and air temperature (AT) as significant variables in the final CCA model (both *p* < 0.05). (**C**) Bar plots showing the relative abundance of aerosol OTUs also detected in local seawater, with points representing average wind speed (red) and wave height (purple) during each sampling period.

Air mass sources were characterized using both 72 h back trajectories and sampling-duration-specific back trajectories (Table S4; Fig. 2A). Geographic trajectory type and oceanic exposure proportion derived from these trajectories were then evaluated in separate PERMANOVA models of bacterial community composition, with season and sampling duration included as additional explanatory factors (Figure S3). PERMANOVA showed that only 72 h geographic trajectory type among the trajectory-derived variables was significantly associated with bacterial community composition (*p* = 0.009; Figure S3A). Season was significant in three of the four models (all *p* < 0.05; Figure S3A,C,D), but not in the model based on 72 h oceanic exposure proportion (*p* = 0.294; Figure S3B). Sampling duration was not significant in any of the four models (all *p* > 0.05; Figure S3). Multivariate dispersion did not differ significantly among the 72 h geographic trajectory groups (permutation test, *p* = 0.189), suggesting this association was less likely to be confounded by differences in dispersion. In contrast, the associations with season should be interpreted more cautiously, as multivariate dispersion differed significantly between seasonal groups (permutation test, *p* = 0.001). These results suggest that, among the trajectory-derived variables examined in this dataset, 72 h geographic trajectory type may be more informative than oceanic exposure proportion in explaining variation in bacterial community composition.

Relationships between bacterial community composition and local environmental variables were explored using canonical correspondence analysis (CCA). The full CCA model including all candidate environmental variables was significant (*p* = 0.004), but variance inflation factors indicated strong collinearity, particularly between seawater temperature (SWT) and air temperature (AT). Forward selection was therefore applied to derive a reduced model. In the final CCA model, AT (*p* = 0.001) and SWT (*p* = 0.003) were identified as significant variables associated with bacterial community composition (Fig. 2B). During spring, SWT and AT ranged from 12.75 to 12.93 °C and 9.31 to 13.22 °C, respectively, whereas in summer they increased to 18.41–20.21 °C and 17.25–19.10 °C (Table S5). However, CCA1 and CCA2 together explained only 16.64% of the total variation (Fig. 2B), indicating that additional factors not captured in the model may also have influenced the observed community variability.

Further examination of the environmental data showed that samples 0528_0800_Q12 and 0812_2105_Q12 were collected under wind speeds below 4 m/s, whereas the other samples ranged from 4.49 m/s to 8.09 m/s (Table S5). Since wind speeds below 4 m/s are less favorable for sea spray aerosol generation, these conditions may contribute to the relatively low overlap with bacteria detected in local seawater in those samples compared with the other samples within their respective trajectory groups, despite their otherwise broadly similar air mass characteristics. This interpretation is consistent with the pattern shown in Fig. 2C. Sample 0812_2105_Q12 from the Ocean group showed no overlap with bacteria detected in local seawater. By contrast, sample 0528_0800_Q12 from the Land_Ocean group showed an intermediate level of overlap within that group, which may have been influenced by the sampler inlet orientation (1°, directly towards the sea) relative to prevailing onshore winds (94.52 ± 30.00°; Table S5), potentially allowing limited inflow of marine air. Despite these low-wind cases, Fig. 2C still indicated that the Ocean group had the highest average overlap with bacteria detected in local seawater (22.74 ± 17.10%, *n* = 6), followed by the Ocean_Land group (18.51 ± 8.91%, *n* = 3), whereas the Land_Ocean group showed the lowest values (4.25 ± 2.16%, *n* = 6).

When testing whether the relative abundance of aerosol OTUs also detected in local seawater was related to environmental factors such as wind speed and wave height, no significant linear correlations were found (all *p* > 0.05; Fig. 2C). This suggests that simple linear models may not adequately capture the factors shaping this overlap in coastal aerosols. Rather, the observed patterns may reflect a combination of marine and meteorological conditions, topography, and the distance traversed by oceanic air masses across land^49,50^. In addition, the limited sample size and temporal variability of the present dataset may have reduced the power to detect such relationships.

### Composition and predicted functional potential of airborne bacterial communities in coastal zones: insights from aerosol taxa overlapping with local seawater taxa

**Fig. 3:**
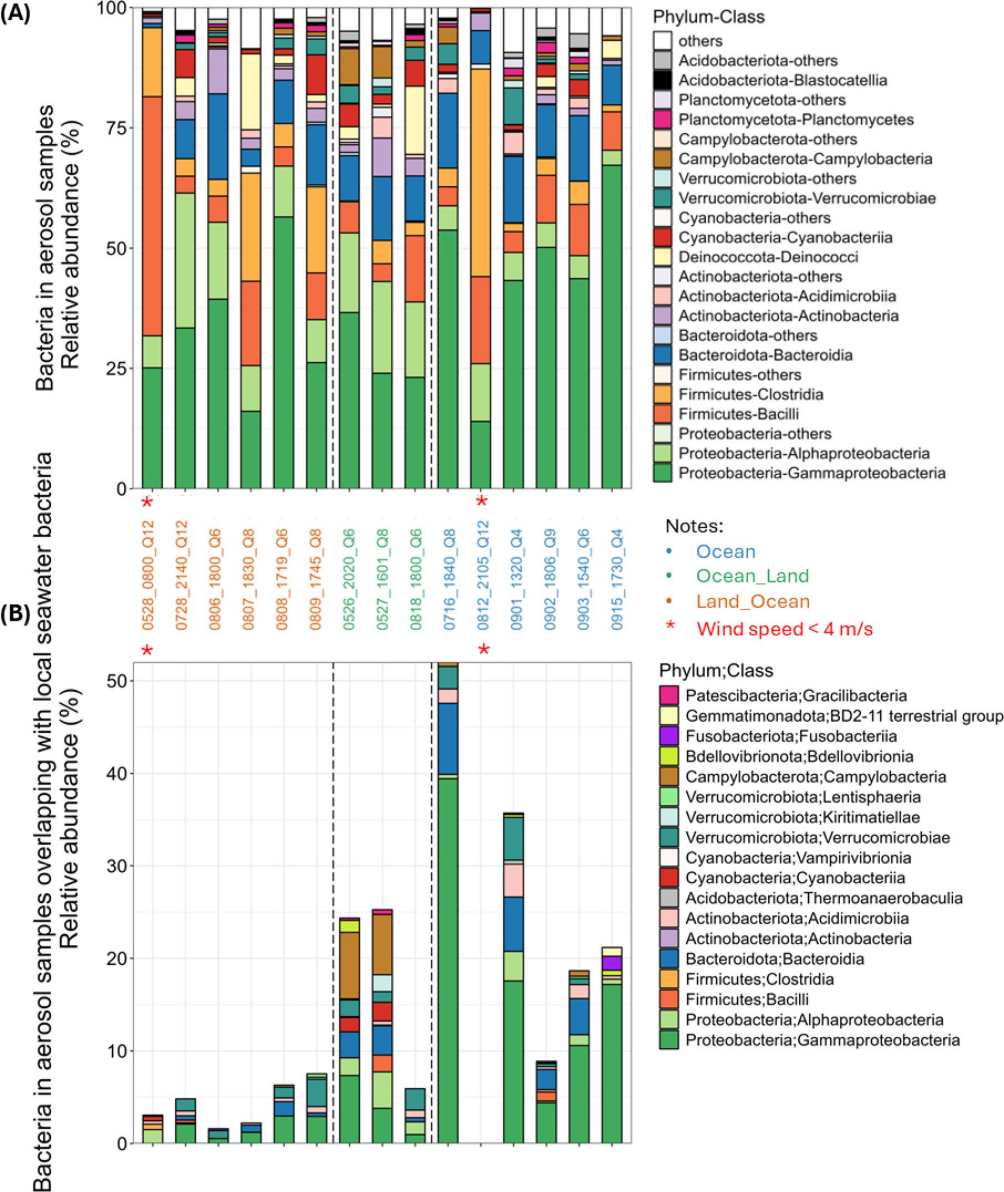
(**A**) Top 10 phyla and their main classes in aerosol samples. (**B**) Classes and their corresponding phyla of bacteria in aerosol samples overlapping with local seawater bacteria. Samples are grouped as Ocean, Ocean_Land, and Land_Ocean based on the geographic types derived from 72 h air mass back trajectories.

The composition of airborne bacterial communities and the aerosol taxa overlapping with bacteria detected in local seawater were visualized at various taxonomic levels (Fig. 3, Figure S4–S6). At the phylum level, *Proteobacteria* (47.96 ± 13.72%), *Firmicutes* (20.36 ± 19.14%), *Bacteroidota* (10.29 ± 4.33%), and *Actinobacteriota* (4.74 ± 3.19%) predominated across samples (Fig. 3A). These findings are consistent with previous studies of airborne bacteria in open seas, coastal regions, and diverse terrestrial environments such as urban, rural, and mountain areas^2,14,16,17,51^. *Firmicutes* dominated in two samples (i.e., 0528_0800_Q12 and 0812_2105_Q12) collected under wind speeds below 4 m/s (63.17 ± 0.01%, *n* = 2) (Fig. 3A). In contrast, *Proteobacteria*, a major phylum in surface seawater and marine aerosols^14,52^, were more abundant in samples collected under wind speeds above 4 m/s (50.89 ± 12.30%, *n* = 13; Fig. 3A). This pattern may reflect reduced sea spray aerosol generation and marine bacterial input under low-wind conditions, although the relationship between wind speed and seawater overlap was not significant (Fig. 2C). These two low-wind samples are retained in the figures for transparency but were not considered in the formal group comparisons described below, because their overlap values were likely influenced not only by trajectory-group patterns but also by sample-specific local factors, including low wind speed in both cases and, for sample 0528_0800_Q12, the orientation of the sampler inlet relative to the prevailing wind direction. At the class level, after excluding the two low-wind samples, the Ocean group showed a higher proportion of *Gammaproteobacteria* (51.61 ± 8.77%, *n* = 5) than the Ocean_Land (27.90 ± 6.16%, *n* = 3) and Land_Ocean (34.30 ± 13.54%, *n* = 5) groups (Wilcoxon test, both *p* < 0.05; Fig. 3A). A considerable proportion of *Gammaproteobacteria* in the Ocean group (34.50 ± 21.88%, *n* = 5) overlapped with taxa detected in local seawater, compared with 13.36 ± 6.71% (*n* = 3) and 6.33 ± 3.19% (*n* = 5) in the Ocean_Land and Land_Ocean groups, respectively (Fig. 3B). Within the Ocean_Land group (*n* = 3), the two spring samples (i.e., 0526_2020_Q6 and 0527_1601_Q8) were separated from the summer sample and from the summer samples in the other groups (Fig. 3B), suggesting that the heterogeneity of this group may largely reflect seasonal differences. Given its small sample size, mixed seasonal composition, and intermediate overlap values, the Ocean_Land group was retained in the figures for exploratory interpretation but was not included in the subsequent formal pairwise comparisons. Accordingly, formal pairwise comparisons were restricted to the Ocean and Land_Ocean groups after exclusion of the two low-wind samples (*n* = 5 per group). For overlap with bacteria detected in local seawater, sensitivity analysis showed that re-including the two low-wind samples attenuated the difference between the Ocean and Land_Ocean groups (*p* = 0.066, compared with *p* = 0.012 when the two low-wind samples were excluded), indicating that this group-wise difference was sensitive to sample inclusion and should be interpreted with caution as an exploratory pattern.

Linear discriminant analysis (LDA) effect size (LEfSe), performed using only the Ocean and Land_Ocean groups after exclusion of the two low-wind samples, identified six taxa enriched in the Ocean group, including the order *Pseudomonadales* and its family *Halieaceae*, the orders *Flavobacteriales* and *Bacteroidales*, and the family *Enterobacteriaceae* together with its affiliated genus *Escherichia-Shigella* (Figure S7A). Given the small group sizes (*n* = 5 per group), these results should be interpreted cautiously. We therefore further examined the relative abundances of these taxa, their overlap with taxa detected in local seawater, and their ecological or aerosolization-related characteristics reported in previous studies to support a cautious interpretation of the LEfSe results.

The family *Halieaceae* accounted for 2.67 ± 1.42% (*n* = 5) in the Ocean group but was detected in only one Land_Ocean sample (0808_1719_Q6; 2.37%) (Figure S5A). Members of this Gram-negative family are commonly found in coastal, open, and deep-sea waters^53^. In this study, a large fraction of *Halieaceae* in the Ocean group (84.10 ± 18.84%, *n* = 5) overlapped with taxa detected in local seawater (Figure S5B). Within *Halieaceae*, the *OM60(NOR5) clade* was the dominant lineage (2.36 ± 1.31%, *n* = 4; Figure S6A), of which 92.81 ± 12.46% overlapped with taxa detected in local seawater (Figure S6B). The *OM60 clade* is widespread in saline environments, particularly in coastal waters^54^, and has been reported in SSAs^55^. Laboratory aerosolization studies further showed its enrichment in the sea surface microlayer (SML) relative to underlying seawater (ULW), although its aerosolization efficiency (the ratio of its fraction in SSAs to that in SML or ULW) was moderate (18% from SML and 16% from ULW)^13^. Within *Pseudomonadales*, the *SAR86 clade* was the most abundant lineage detected, accounting for 10.74 ± 9.45% (*n* = 5) in the Ocean group compared with 0.97 ± 0.71% (*n* = 5) in the Land_Ocean group (Figure S5A). *SAR86* is a well-known oligotrophic marine lineage that is often abundant in surface waters and plays a crucial role in marine carbon and sulfur cycling^56^. It was not identified as a LEfSe-enriched taxon here, possibly due to high within-group variance driven by one Ocean sample with very low abundance (0915_1730_Q4: 0.13% vs. 13.39 ± 8.74% in the others (*n* = 4)). Previous studies have reported *SAR86* enrichment in the SML and high aerosolization potential (491% from SML; 352% from ULW)^13^, although its presence in aerosols has not been reported consistently^15^.

The order *Flavobacteriales* accounted for 5.29 ± 2.46% (*n* = 5) in the Ocean group and 2.41 ± 1.46% (*n* = 5) in the Land_Ocean group (Figure S4A). At the family level, *Flavobacteriaceae* (2.45 ± 0.01%, *n* = 5) and the *NS9 group* (1.67 ± 1.34%, *n* = 4) were the main families detected within *Flavobacteriales* in the Ocean group, whereas *Flavobacteriaceae* (1.58 ± 1.25%, *n* = 4) was the main family detected in the Land_Ocean group, with only one sample (0809_1745_Q8: 1.42%) containing the *NS9 group* (Figure S5A). In this study, *Flavobacteriaceae* comprised diverse genera across samples and was therefore not discussed further. For the *NS9 group*, 77.61 ± 33.49% (*n* = 4) overlapped with taxa detected in local seawater (Figure S5B). Members of the *NS9 group* typically inhabit coastal waters and have been reported to exhibit high aerosolization potentials from both the SML (91%) and ULW (84%)^13,57^.

The order *Bacteroidales* was detected in all Ocean group samples (2.27 ± 1.59%, *n* = 5) but in only one Land_Ocean sample (0808_1719_Q6: 0.17%). Although some members of *Bacteroidales* are well-known components of the human microbiome^58^, this order has rarely been reported in marine or coastal aerosols and has rarely been detected in surface seawater^59^. In the Ocean group, *Bacteroidales* were mainly represented by *Marinifilaceae* (1.08 ± 0.86%, *n* = 5) and *Prolixibacteraceae* (0.85 ± 0.64%, *n* = 4), of which 55.45 ± 18.80% and 53.05 ± 33.54%, respectively, overlapped with taxa detected in local seawater (Figure S5B). Both families have been reported in marine environments, particularly coastal sediments^60^.

The family *Enterobacteriaceae* and the genus *Escherichia-Shigella* were detected in 4 of 5 Ocean group samples (12.60 ± 15.99% and 12.45 ± 16.09%, respectively), but were absent from the Land_Ocean group (Figure S5A, Figure S6A). Because species-level discrimination within the *Escherichia/Shigella* group remains challenging based on 16S rRNA gene sequences alone, especially for closely related taxa, these sequences are conservatively reported here as *Escherichia-Shigella*. Members of this group are commonly associated with fecal contamination and sewage-impacted environments. *Escherichia-Shigella* was not detected in the local seawater samples collected in summer 2018^37^. Although this comparison should be interpreted cautiously because the seawater and aerosol samples were collected in different years, the detection of *Escherichia-Shigella* in the 2021 summer aerosol samples may reflect episodic contamination of coastal waters, beach sands, or nearby air by human activities, seabirds, or wastewater inputs. Relevant coastal water monitoring data from Ostend—Dunes and Sea (~ 953 m from the aerosol sampling site) recorded elevated *Escherichia coli* (*E. coli*) counts near the aerosol sampling dates: 860 CFU/100 mL on August 23 for samples 0901_1320_Q4, 0902_1806_Q8.9, and 0903_1540_Q6, and 444 CFU/100 mL on September 10 for the sample 0915_1730_Q4, compared with values below 100 CFU/100 mL on other dates (Table S8). Similar enrichment of fecal-associated bacteria, such as *E. coli*, from seawater into the air after sewage discharge has been reported in the Bay of Gdańsk^61,62^, and laboratory mesocosm experiments simulating phytoplankton blooms reported aerosolization factors of 155%-569%^10^. Together, these observations suggest that episodic fecal-associated inputs to coastal aerosols may occur under certain conditions. However, the present study does not allow assessment of viability, infectivity, or the presence of specific pathogens. Future work should therefore examine whether such events are accompanied by other fecal-associated or potentially pathogenic microorganisms, as well as their abundance and viability in coastal aerosols.

To explore the predicted functional potential of airborne bacterial communities, we used PICRUSt2 to infer metabolic pathways from 16S rRNA gene sequences. NSTI values were generally low across the sequences used for prediction (mean ± SD: 0.08 ± 0.04), indicating that the PICRUSt2 predictions were reasonably well supported, although they should still be interpreted cautiously. Level 3 pathways with relative abundances > 0.1% that differ significantly between the Ocean and Land_Ocean groups are shown in Figure S7B,C. The Land_Ocean group showed higher predicted representation of pathways related to bacterial chemotaxis, which is associated with environmental sensing and motility, and to terpenoid and steroid biosynthesis, a function often linked to plant-associated microbes. Higher predicted representation of pathways related to xenobiotic metabolism, including drug metabolism via cytochrome P450 and other enzymes, was also observed in this group, which may indicate a broader potential for transforming structurally diverse external organic compounds rather than direct relevance to specific pollutants. However, KEGG pathways annotated under ‘human disease’ categories (e.g., chemical carcinogenesis, amyotrophic lateral sclerosis, fluid shear stress, and atherosclerosis) are not interpreted here in relation to the sampled aerosol environment, as these labels reflect database classification conventions rather than direct disease relevance. In contrast, the Ocean group exhibited higher predicted representation of pathways related to protein export and diverse metabolic activities involving carbohydrates, fatty acids, cofactors, and vitamins (e.g., riboflavin and vitamin B6), as well as the biosynthesis of lipopolysaccharides and secondary metabolites such as monobactam. Overall, these exploratory results suggest differences in predicted functional potential between the two groups, with the Land_Ocean group showing relatively greater representation of pathways related to environmental sensing and xenobiotic-associated metabolism, and the Ocean group showing relatively greater representation of pathways related to protein export and diverse metabolic processes.

### Exploring the environmental affiliations of bacteria overlapping with local seawater bacteria in aerosol samples

The distinct compositions and predicted functional potential of airborne bacterial communities in the Land_Ocean and Ocean groups suggest that bacteria associated with local seawater and potentially transferred via sea spray aerosols may contribute to coastal airborne bacterial communities. However, their persistence in the atmosphere and adaptation to atmospheric conditions remain poorly understood. To further characterize their environmental affiliations, we used ProkAtlas^47^ to predict potential habitat affiliations based on 16S rRNA gene sequences. Aerosol samples from the Ocean group showed predominant affiliation with ocean-related habitats (e.g., marine, salt marsh, seawater), whereas those from the Land_Ocean group were associated with a broader range of terrestrial environments, including soil- and plant-related habitats, as well as wastewater-related habitats (Figure S8A). These patterns are consistent with Zhao et al. (2022), who reported that offshore airborne bacteria are primarily linked to marine sources, while urban air harbors taxa associated with terrestrial and anthropogenic settings^51^. Such contrasting habitat affiliations likely reflect the interaction between marine and terrestrial air masses in coastal zones. Bacteria in aerosol samples that were also detected in local seawater showed strong affiliation with ocean-related habitats, followed by freshwater and other aquatic environments (Figure S8B), suggesting that these taxa are mainly associated with aquatic habitats. However, which taxa are most capable of aerosolization and prolonged atmospheric persistence remains uncertain. Previous studies have shown that aerosolization of marine bacteria is strain-specific and influenced by cellular properties (e.g., cell size, shape, and membrane composition) and environmental factors such as seawater biology and wind speed^10,11^. In this study, more than half of the bacteria in Ocean-group aerosol samples that overlapped with local seawater bacteria (53.70 ± 0.07%, *n* = 5) remained unidentified at the genus level (Figure S6), underscoring the need for further characterization of these taxa.

### Study limitations

There are several limitations of this study that should be acknowledged. First, this was a coastal case study based on a limited number of aerosol samples, which constrained statistical power and the strength of group-wise inference. Some subgroup analyses also involved small sample sizes, and the restricted comparison between the Ocean and Land_Ocean groups was sensitive to the inclusion or exclusion of the two low-wind samples. These group-wise patterns should therefore be interpreted cautiously as exploratory findings. Second, the aerosol samples and the local seawater reference dataset were collected in different years. Accordingly, the observed overlap should be interpreted as similarity to a seasonal local reference community rather than definitive evidence of direct source origin, as interannual variability may have affected the degree of overlap. Third, the low biomass of the aerosol samples required 40 PCR cycles to obtain sufficient amplicons for sequencing. Although control filters helped assess low-level contamination, they could not assess potential PCR-related amplification bias, and preferential amplification of some taxa therefore cannot be excluded. Fourth, predicted functional profiles were inferred from Nanopore-based full-length 16S rRNA gene data using PICRUSt2 and thus represent predicted functional potential rather than direct measurements of gene content or activity. Although read filtering, error correction, and NSTI-based filtering were applied, residual sequencing errors and phylogenetic placement uncertainty may still have affected downstream pathway predictions. Fifth, this study did not assess viability, infectivity, or atmospheric persistence directly. Together, these limitations highlight the need for future studies with larger sample sets, concurrent seawater and aerosol sampling, improved low-biomass sampling and molecular workflows that reduce reliance on extensive PCR amplification, direct assessment of aerosolized microbial viability and persistence, and complementary approaches beyond marker-gene sequencing.

## Conclusion

In this coastal case study, variation in airborne bacterial communities was associated with interacting marine and terrestrial influences. Air mass origin and environmental conditions were associated with differences in community composition. Samples influenced by oceanic air masses, particularly under conditions favorable for sea spray aerosol generation, tended to show greater overlap with bacteria detected in local seawater. These samples also tended to differ in taxonomic composition and predicted functional potential from samples under weaker marine influence. However, these group-wise patterns were based on a small number of samples and were sensitive to the inclusion or exclusion of the two low-wind samples. They should therefore be regarded as exploratory findings rather than definitive evidence of marine influence. Most bacteria in aerosol samples overlapping with local seawater bacteria showed strong affiliations with ocean-related environments, suggesting that these taxa were mainly associated with aquatic habitats. Their presence in coastal air may reflect physical transfer through sea spray aerosol generation, although their persistence and activity in the atmosphere remain uncertain. Future studies should therefore investigate the viability, persistence, and atmospheric behavior of marine-associated airborne bacteria, while improving taxonomic and functional characterization through approaches beyond marker-gene inference. Such work would help clarify their ecological roles and potential relevance to atmospheric processes in coastal environments.

## Supplementary Information

Below is the link to the electronic supplementary material.

Supplementary Material 1

## Data Availability

The sequencing data generated in this study are publicly available in the European Nucleotide Archive (ENA) repository under the project accession numbers PRJEB106530 and PRJEB106579.
