# Supplementary material for: Marine influence on airborne bacterial community composition and predicted functional potential in coastal zones: a case study from Ostend, Belgium
Source: Sci Rep. 2026 May 22;16:23505. doi: 10.1038/s41598-026-54664-0 (PMC13416136; doi:10.1038/s41598-026-54664-0)
Supplement: Supplementary file 1 — Supplementary Material 1 [file 41598_2026_54664_MOESM1_ESM.docx]

**Supplementary information**

**Marine influence on airborne bacterial community composition and predicted functional potential in coastal zones: a case study from Ostend, Belgium**

Yunmeng Li^1,2,3^, Zixia Liu^2^, Pascal I. Hablützel^1,4^, Wannes Van Beeck^3^, Colin R. Janssen^2^, Sarah Lebeer^3^, Jana Asselman^2^, Maarten De Rijcke^1,*^

^1^Flanders Marine Institute (VLIZ), InnovOcean Campus, Jacobsenstraat 1, 8400 Ostend, Belgium

^2^Blue Growth Research Lab, Ghent University, Wetenschapspark 1, 8400 Ostend, Belgium

^3^Laboratory of Applied Microbiology and Biotechnology, Department of Bioscience Engineering, University of Antwerp, Groenenborgerlaan 171, 2020 Antwerp, Belgium

^4^Department of Biology, Vrije Universiteit Brussel, Pleinlaan 2, 1050 Brussels, Belgium

^*^ Corresponding author at: Flanders Marine Institute (VLIZ), InnovOcean Campus, Jacobsenstraat 1, 8400 Ostend, Belgium.

E-mail address: [maarten.de.rijcke@vliz.be](mailto:maarten.de.rijcke@vliz.be) (M. De Rijcke).

**Summary: Total 26 pages, 8 tables, and 8 figures**

**Contents**

**Table S1.** Overview of studies published since 2010 on low-altitude airborne bacterial communities over open seas and coastal zones using high-throughput sequencing techniques 3

**Table S2.** Features of glass fiber (EPM2000) and quartz fiber (QM-A) filters 7

**Table S3.** Aerosol sample information, including sample ID, sampling date and time, and total collected air volume 8

**Table S4.** Sample groups categorized by geographic type and proportion of time spent over oceanic regions based on 72 h and sampling-duration-specific air mass back trajectories 10

**Table S5.** Average environmental conditions for each aerosol sample 11

**Table S6.** Conditions of DNA extraction, full-length 16S rRNA gene amplification, and Nanopore sequencing for each aerosol sample 12

**Table S7.** Grouping of habitat categories used in the ProkAtlas analysis 14

**Table S8.** Water quality monitoring data for coastal waters at Ostend – Dunes and Sea (~953 m from the aerosol sampling site) during the aerosol sampling period 15

**Figure S1.** (A) Map of the Marine Station Ostend showing the aerosol sampling site and nearby environmental monitoring stations, and (B,C) photographs of the rooftop sampling setup 16

**Figure S2.** Rarefaction curves for each aerosol sample 17

**Figure S3.** PCoA plots and PERMANOVA results showing the effects of geographic trajectory type and oceanic exposure proportion, derived from 72 h and sampling-duration-specific back trajectories, on airborne bacterial community composition 18

**Figure S4.** (A) Top 20 orders of bacteria in aerosol samples. (B) Top 20 orders of bacteria in aerosol samples overlapping with local seawater bacteria 19

**Figure S5.** (A) Top 30 families of bacteria in aerosol samples. (B) Top 30 families of bacteria in aerosol samples overlapping with local seawater bacteria 20

**Figure S6.** (A) Top 30 genera of bacteria in aerosol samples. (B) Top 30 genera of bacteria in aerosol samples overlapping with local seawater bacteria 21

**Figure S7.** Differential (A) taxa identified by LEfSe and (B,C) predicted metabolic functions inferred by PICRUSt2 between the Ocean and the Land_Ocean groups 22

**Figure S8.** Preferential habitats inferred by ProkAtlas for (A) bacteria in aerosol samples and (B) bacteria in aerosol samples overlapping with local seawater bacteria 23

**References**

**Table S1.** Overview of studies published since 2010 on low-altitude airborne bacterial communities over open seas and coastal zones using high-throughput sequencing (HTS) techniques. Relevant papers were identified through searches in databases including Google Scholar and Web of Science using the following terms and Boolean operations: (“airborne bacteria AND sequencing” OR “airborne bacterial communities”) AND (“sea spray aerosols” OR “marine aerosols” OR “bioaerosols AND outdoor” OR “coast” OR “open sea”).

| Sampling sites | Sampling methods and sequencing techniques | References |
| --- | --- | --- |
| **Open sea** |  |  |
| Upper deck of the Research Vessel *Xue Long* across the western Pacific Ocean, the northern Pacific Ocean, the Arctic Ocean, and the Norwegian Sea | - Filtration: 25-mm diameter and 1.0-μm pore-size glass fiber filters & 1,000 L/min for 9-14 days per sample - NGS: 16S rRNA gene (V3-V4) amplicon sequencing using 454 pyrosequencing | (Xia et al., 2015) |
| 10 m asl, from the North Sea to the Baltic Sea via Skagerrak and Kattegat | - Liquid impingement: a Dycor XMX/2L MIL impinger with 5 mL sterile PBS & 12 L/min for 1 h per sample - NGS: 16S rRNA gene (V1-V5) amplicon sequencing using 454 pyrosequencing (*! Rarefaction curves didn’t reach a plateau for all samples*) | (Seifried et al., 2015) |
| 15 m asl, the Bohai Sea, the Yellow Sea, and northwestern Pacific Ocean | - Filtration & Impaction: a six-stage cascade impactor with 0.22-μm pore-size PC membrane filters & 28.3 L/min for 1 h per sample - NGS: 16S rRNA gene (V3-V4) amplicon sequencing using Illumina HiSeq 2500 | (Ma et al., 2019) |
| At the front of the ship, an East-West transect of the entire Mediterranean Sea | - Filtration: Whatman 41 filters (20-μm pore-size cellulose filter) & 700 L/min for 24 h per sample & for each sample, subsamples (2 cm x 2 cm) were used for DNA extraction (performed in triplicate) and then pooled into one sample for sequencing. - NGS: 16S rRNA gene (V4) amplicon sequencing using Illumina MiSeq | (Mescioglu et al., 2019) |
| 59 m asl, Amsterdam-Island; 37 m asl, Station-Nord, Greenland. | - Filtration: quartz fiber filters & one week for total air volume of 4.4-5.3 x 10^3^ m^3^ per filter & 3 circular pieces (diameter of one piece:38 mm) of each sample was used for sequencing - NGS: 16S rRNA gene (V3-V4) amplicon sequencing using Illumina MiSeq | (Tignat-Perrier et al., 2019) |
| 23 m asl, the Southern Ocean | - Filtration: 47-mm diameter and 0.4-μm pore-size PC membrane filters & 39 L/min for around 21.58 h per sample - NGS: 16S rRNA gene (V4-V5) amplicon sequencing using Illumina MiSeq | (Uetake et al., 2020) |
| 25 m asl, the Great Barrier Reef marine ecosystem | - Liquid cyclonic impingement: a Coriolis air sampler with 15 mL PBS & 300 L/min for 0.5-6 h per sample - NGS: 16S rRNA gene (V3-V4) amplicon sequencing using Illumina MiSeq *(27 of 53 air samples passed sequencing*) | (Archer et al., 2020) |
| 30 m asl, marine regions along the Antarctic coast | - Filtration: 20.3 x 25.4 cm quartz microfiber filters & 1,050 L/min for 24 h per sample & for each sample, six pieces of film were randomly collected with a hole punch and then used for DNA extraction and sequencing. - NGS: 16S rRNA gene (V3-V4) amplicon sequencing using Illumina MiSeq | (Cao et al., 2021) |
| 15 or 30 m asl, the north Atlantic and Pacific Oceans | - Filtration: 47-mm diameter 0.45-μm pore-size PVDF filters & 20 or 30 L/min for 12-24 h per sample & DNA was extracted from three sample replicates separately, and their PCR products were pooled in one for sequencing. - NGS: 16S rRNA gene (V4-V5) amplicon sequencing using Illumina MiSeq | (Lang-Yona et al., 2022) |
| 15 m asl, from the Chinese marginal seas to the northwestern Pacific Ocean | - Filtration & Impaction: a six-stage cascade impactor with 0.22-μm pore-size PC membrane filters & 28.3 L/min for 12 h - NGS: 16S rRNA gene (V3-V4) amplicon sequencing using Illumina NovaSeq PE250 | (Zhang et al., 2023) |
| 19 m asl, the Northern Pacific Ocean during a cruise covering 7724 km between 37°N 126°E and 58°N 179°E (along transects from Korea to the Bering Sea). | - Filtration: quartz filters collecting PM2.5 & 1,000 L/min for 21-24 h per sample - NGS: 16S rRNA gene (V4-V5) amplicon sequencing using Illumina MiSeq | (Jang et al., 2024) |
| 10 m asl, the Pacific Ocean | - Dry cyclonic impaction: A Coriolis Compact & 50 L/min for 12 h per sample - NGS: 16S rRNA gene (V4-V5) and 16S rRNA (V4-V5, cDNA) amplicon sequencing using Illumina Miniseq | (Tastassa et al., 2025) |
| **Coastal zones** |  |  |
| Just above the high tide line, three beaches in central California, USA | - Filtration: 25-mm 3.0-μm pore-size PTFE filters & 10 L/min for 4 h per sample - NGS: 16S rRNA gene (V4) amplicon sequencing using Illumina MiSeq | (Graham et al., 2018) |
| 458 m agl, the tallest communication tower (about 10 km inland) in Tokyo, Japan | - Filtration: 47-mm quartz fiber filters: 15 L/min for 48-72 h per sample - NGS: 16S rRNA gene (V3-V4) amplicon sequencing using Illumina MiSeq | (Uetake et al., 2019) |
| 1.5 m above the building roof (over 20 m agl, about 600 m inland) in Weihai, China | - Filtration: PM2.5 collected by 0.45-μm pore-size quartz fiber filters & 100 L/min for 23 h per sample - NGS: 16S rRNA gene (V3-V4) amplicon sequencing using Illumina MiSeq | (Wei et al., 2020) |
| Roof of a teaching building (~9 m agl, ~7 km from sea) on the Laoshan campus of Ocean University in China | - Filtration & Impaction: a six-stage cascade impactor with 0.22-μm pore-size PC membrane filters & 28.3 L/min for 30 min per sample - NGS: 16S rRNA gene (V3-V4) amplicon sequencing using Illumina MiSeq | (Ma et al., 2021) |
| 5 or 20 m asl, Imperial beach (IB) and Scripps Institution of Oceanography (within 100 m from the coastline) | - Filtration: 47-mm diameter quartz fiber filters & 30 L/min for 22 h per sample & 1/4 of each sample was used for sequencing - NGS: 16S rRNA gene (V4) amplicon sequencing using Illumina MiSeq | (Pendergraft et al., 2021) |
| 12 m asl with a distance of 2 km from the sea, Savona, Italy. | - Filtration: 47-mm diameter QM-A quartz filters & 38.6 L/min for 24 h per sample - NGS: 16S rRNA gene (V3-V4) amplicon sequencing using Illumina MiSeq | (Palladino et al., 2021) |
| 0.8 m above the beach sand within the intertidal zone at the Darwin Beach, Miami, Florida, USA | - Filtration: 47-mm diameter 0.45-μm pore size cellulose membrane filters & 12 L/min for 3 h per sample - NGS: 16S rRNA gene (V4) amplicon sequencing using Illumina MiSeq (*! some samples didn’t pass sequencing due to low DNA concentrations*) | (Abdool-Ghany et al., 2023) |
| 15 m agl, roof top of a building at the national air sampling station of Shandong University (1-2 km inland), Weihai, China | - Filtration: PM2.5 collected by 47-mm diameter quartz fiber filters: unknown sampling flow rate for 24 h - NGS: 16S rRNA gene (V3-V4) amplicon sequencing using Illumina MiSeq | (He et al., 2024) |
| Huaniao island located in the offshore East China Sea | - Filtration: 203 × 254 mm quartz microfiber filters & 1,050 L/min for unknown duration & two 1/8 sample filters were used for DNA extraction and sequencing - NGS: 16S rRNA gene (V3-V4) amplicon sequencing using Illumina NovaSeq PE250 | (Wang et al., 2024) |

Note: asl, above sea level; agl, above ground level; NGS, next-generation sequencing; PBS, phosphate-buffered saline; PC, polycarbonate; PTFE, polytetrafluoroethylene; PVDF, polyvinylidene fluoride or polyvinylidene difluoride.

**Table S2.** Features of glass fiber (EPM2000) and quartz fiber (QM-A) filters as described by the manufacturer.

| Features | EPM2000  (Whatman, 1882-047) | QM-A  (Whatman, 1851-047) |
| --- | --- | --- |
| Diameter (mm) | 47 | 47 |
| Pore size (µm) | NA | 2.2 |
| 0.3 µm particle retention (%) | 99.95 | 99.95 |
| Thickness (µm) | 450±15 | 450±15 |
| Flow rate (m^3^/min) | 1.52 | 1.1325 |
| Filter media | Glass fiber | High-purity quartz microfiber |
| Particles | PM10 | PM10 |
| Chemical analysis | 100% pure borosilicate glass of special purity enabling detailed chemical analysis of trace pollutants to take place with the minimum of interference or background. | High-purity (SiO_2_) microfiber filters are used for air sampling in acidic gases, stacks, flues and aerosols, particularly at high temperatures up to 800 °C and in PM10 testing. Due to the low levels of alkaline earth metals, “artifact” products of sulfates and nitrates (from SO_2_ and NO_2_) are virtually eliminated. |

References:

1) Whatman® EPM2000 air sampling filters diam. 47 mm (sigmaaldrich.com) (link: <https://www.sigmaaldrich.com/DE/de/product/aldrich/wha1882047>),

2) Whatman® QM-A-Quarzfaserfilter Circles, 47 mm 100/pk | Sigma-Aldrich (sigmaaldrich.com) (link: <https://www.sigmaaldrich.com/DE/de/product/aldrich/wha1851047>),

3) Whatman Gr. QM-A Quartz Air Sampling Filter, 47mm, 100 Pack (scientificfilters.com) (link: <https://scientificfilters.com/filter-papers-glass-microfiber-filters-quartz-1851-047>).

**Table S3.** Aerosol sample information, including sample ID, sampling date and time, and total collected air volume.

- Aerosol samples were collected on the rooftop of Warehouse 4 at the Marine Station Ostend (MSO; 51°14′10′′N, 2°55′42′′E; approximately 300 m from the coastline) in Ostend, Belgium, using an AirCube air sampler. The sampling air flow rate through each filter was approximately 22 L/min, as measured with a rotameter. The total air volume collected for each sample was calculated by multiplying the sampling duration by 22 L/min.
- Recorded dates and times are given in Belgian local time (CEST). Seasons were defined based on astronomical equinoxes and solstices for the year 2021: March 20 to June 20 for spring, and June 21 to September 21 for summer.
- Sample IDs were formatted as “sampling end date_end time_filter type (Q for QM-A; E for EPM2000) and sampling duration”.

| Sample ID | Season | Start date | Start time | End date | End time | Duration (h) | Total air volume (m^3^) | Filter type |
| --- | --- | --- | --- | --- | --- | --- | --- | --- |
| 0525_1222_E1 | Spring | 20210525 | 11:22 | 20210525 | 12:22 | 1 | 1.32 | EPM2000 |
| 0525_1222_Q1 | Spring | 20210525 | 11:22 | 20210525 | 12:22 | 1 | 1.32 | QM-A |
| 0525_1530_E2 | Spring | 20210525 | 13:30 | 20210525 | 15:30 | 2 | 2.64 | EPM2000 |
| 0525_1530_Q2 | Spring | 20210525 | 13:30 | 20210525 | 15:30 | 2 | 2.64 | QM-A |
| 0525_1925_E3 | Spring | 20210525 | 16:25 | 20210525 | 19:25 | 3 | 3.96 | EPM2000 |
| 0525_1925_Q3 | Spring | 20210525 | 16:25 | 20210525 | 19:25 | 3 | 3.96 | QM-A |
| 0526_1325_E4 | Spring | 20210526 | 09:25 | 20210526 | 13:25 | 4 | 5.28 | EPM2000 |
| 0526_1325_Q4 | Spring | 20210526 | 09:25 | 20210526 | 13:25 | 4 | 5.28 | QM-A |
| 0526_2020_E6 | Spring | 20210526 | 14:20 | 20210526 | 20:20 | 6 | 7.92 | EPM2000 |
| 0526_2020_Q6 | Spring | 20210526 | 14:20 | 20210526 | 20:20 | 6 | 7.92 | QM-A |
| 0527_1601_E8 | Spring | 20210527 | 08:01 | 20210527 | 16:01 | 8 | 10.56 | EPM2000 |
| 0527_1601_Q8 | Spring | 20210527 | 08:01 | 20210527 | 16:01 | 8 | 10.56 | QM-A |
| 0528_0800_E12 | Spring | 20210527 | 20:00 | 20210528 | 08:00 | 12 | 15.84 | EPM2000 |
| 0528_0800_Q12 | Spring | 20210527 | 20:00 | 20210528 | 08:00 | 12 | 15.84 | QM-A |
| 0529_1452_E24 | Spring | 20210528 | 14:52 | 20210529 | 14:52 | 24 | 31.68 | EPM2000 |
| 0529_1452_Q24 | Spring | 20210528 | 14:52 | 20210529 | 14:52 | 24 | 31.68 | QM-A |
| 0531_0840_E36 | Spring | 20210529 | 20:40 | 20210531 | 08:40 | 36 | 47.52 | EPM2000 |
| 0531_0840_Q36 | Spring | 20210529 | 20:40 | 20210531 | 08:40 | 36 | 47.52 | QM-A |
| 0602_1013_E48 | Spring | 20210531 | 10:13 | 20210602 | 10:13 | 48 | 63.36 | EPM2000 |
| 0602_1013_Q48 | Spring | 20210531 | 10:13 | 20210602 | 10:13 | 48 | 63.36 | QM-A |
| 0605_2109_E58.7 | Spring | 20210603 | 10:27 | 20210605 | 21:09 | 58.7 | 77.484 | EPM2000 |
| 0605_2109_Q58.7 | Spring | 20210603 | 10:27 | 20210605 | 21:09 | 58.7 | 77.484 | QM-A |
| 0611_0950_E72 | Spring | 20210608 | 09:50 | 20210611 | 09:50 | 72 | NA (missing) | EPM2000 |
| 0611_0950_Q72 | Spring | 20210608 | 09:50 | 20210611 | 09:50 | 72 | 95.04 | QM-A |
| 0716_1840_Q8 | Summer | 20210716 | 09:40/14:40 | 20210716 | 13:40/18:40 | 8 | 10.56 | QM-A |
| 0728_2140_Q12 | Summer | 20210728 | 09:40 | 20210728 | 21:40 | 12 | 15.48 | QM-A |
| 0806_1800_Q6 | Summer | 20210806 | 11:00/15:00 | 20210806 | 14:00/18:00 | 6 | 7.92 | QM-A |
| 0807_1830_Q8 | Summer | 20210807 | 10:30 | 20210807 | 18:30 | 8 | 10.56 | QM-A |
| 0808_1719_Q6 | Summer | 20210808 | 11:19 | 20210808 | 17:19 | 6 | 7.92 | QM-A |
| 0809_1745_Q8 | Summer | 20210809 | 9:45 | 20210809 | 17:45 | 8 | 10.56 | QM-A |
| 0812_2105_Q12 | Summer | 20210812 | 9:05 | 20210812 | 21:05 | 12 | 15.48 | QM-A |
| 0818_1800_Q6 | Summer | 20210818 | 12:00 | 20210818 | 18:00 | 6 | 7.92 | QM-A |
| 0901_1320_Q4 | Summer | 20210901 | 9:20 | 20210901 | 13:20 | 4 | 5.28 | QM-A |
| 0902_1806_Q8.9 | Summer | 20210902 | 9:12 | 20210902 | 18:06 | 8.9 | 11.748 | QM-A |
| 0903_1540_Q6 | Summer | 20210903 | 9:40 | 20210903 | 15:40 | 6 | 7.92 | QM-A |
| 0915_1730_Q4 | Summer | 20210915 | 13:30 | 20210915 | 17:30 | 4 | 5.28 | QM-A |

**Table S4.** Sample groups categorized by geographic type and proportion of time spent over oceanic regions based on 72 h and sampling-duration-specific air mass back trajectories.

- ‘Ocean’, representing trajectories passing almost entirely (≈100%) over the ocean; ‘Land’, representing trajectories passing almost entirely (≈100%) over land; ‘Ocean_Land’, representing trajectories crossing both ocean and land with the ocean closer to the sampling site; and ‘Land_Ocean’, representing trajectories crossing both ocean and land with land closer to the sampling site.
- Groups based on oceanic proportions were defined by 10% intervals.

| Samples | 72 h back trajectories | | Sampling-duration-specific trajectories | |
| --- | --- | --- | --- | --- |
|  | Geographic types | Oceanic proportions (%) | Geographic types | Oceanic proportions (%) |
| 0526_2020_Q6 | Ocean_Land | 56.25 -> [50,60) | Ocean | 100 -> [90,100] |
| 0527_1601_Q8 | Ocean_Land | 74.17 -> [70,80) | Ocean | ~90 -> [90,100] |
| 0528_0800_Q12 | Land_Ocean | 85.41 -> [80,90) | Land | <20 -> [10,20) |
| 0716_1840_Q8 | Ocean | 100 -> [90,100] | Ocean | 100 -> [90,100] |
| 0728_2140_Q12 | Land_Ocean | 94.17 -> [90,100] | Land_Ocean | ~67 -> [60,70) |
| 0806_1800_Q6 | Land_Ocean | 95.83 -> [90,100] | Land_Ocean | 70 -> [70,80) |
| 0807_1830_Q8 | Land_Ocean | 70.83 -> [70,80) | Land_Ocean | ~32 -> [30,40) |
| 0808_1719_Q6 | Land_Ocean | 94.58 -> [90,100] | Land_Ocean | ~55 -> [40,50) |
| 0809_1745_Q8 | Land_Ocean | 82.5 -> [80,90) | Land_Ocean | 62.5 -> [60,70) |
| 0812_2105_Q12 | Ocean | 100 -> [90,100] | Ocean | 100 -> [90,100] |
| 0818_1800_Q6 | Ocean_Land | 62.08 -> [60,70) | Ocean | 100 -> [90,100] |
| 0901_1320_Q4 | Ocean | 99.97 -> [90,100] | Ocean | 100 -> [90,100] |
| 0902_1806_Q8.9 | Ocean | 100 -> [90,100] | Ocean | 100 -> [90,100] |
| 0903_1540_Q6 | Ocean | 70 -> [70,80) | Ocean_Land | ~67 -> [60,70) |
| 0915_1730_Q4 | Ocean | 100 -> [90,100] | Ocean | 100 -> [90,100] |

**Table S5.** Average environmental conditions for each aerosol sample (mean ± standard deviation).

- SWT, seawater temperature; WH, wave height; AT, air temperature; RH, relative humidity; SR, solar radiation; WS, wind speed; Precip., precipitation; WD, wind direction.
- * For sample 0716_1840_Q8, the primary wind directions during the sampling period were 1° and 353°.
- Ocean variables were monitored at 30 min intervals by the Buoy, while meteorological factors were recorded at 10 min intervals by the Weather station.

| Sample ID | SWT (℃) | WH (cm) | AT (℃) | RH (%) | SR (W/m^2^) | WS (m/s) | Precip. (mm) | WD (°) |
| --- | --- | --- | --- | --- | --- | --- | --- | --- |
| 0525_1222_E1 & Q1 | 12.37±0.05 | 106.3±5.4 | 11.85±0.15 | 77.83±0.69 | 551.17±137.32 | 8.23±0.56 | 0.00±0.00 | 257.33±2.92 |
| 0525_1530_E2 & Q2 | 12.68±0.07 | 93.0±3.5 | 12.23±0.41 | 75.62±3.29 | 540.00±195.55 | 8.57±0.86 | 0.00±0.00 | 262.08±2.09 |
| 0525_1925_E3 & Q3 | 12.43±0.05 | 90.0±5.4 | 11.56±0.46 | 82.72±2.26 | 155.50±59.68 | 7.58±1.02 | 0.03±0.06 | 255.06±6.32 |
| 0526_1325_E4 & Q4 | 12.42±0.10 | 60.0±5.7 | 11.28±0.36 | 80.83±2.30 | 401.29±163.99 | 6.70±0.91 | 0.00±0.00 | 283.00±5.44 |
| 0526_2020_E6 & Q6 | 12.79±0.11 | 74.4±5.7 | 12.82±0.58 | 75.14±3.31 | 501.00±322.80 | 8.09±0.76 | 0.00±0.00 | 268.16±8.09 |
| 0527_1601_E8 & Q8 | 12.75±0.13 | 76.4±12.3 | 12.25±0.34 | 81.14±4.43 | 419.69±197.31 | 6.01±0.69 | 0.00±0.00 | 318.94±18.24 |
| 0528_0800_E12 & Q12 | 12.93±0.10 | 57.3±5.0 | 9.31±1.03 | 85.68±4.64 | 25.95±51.78 | 2.21±1.06 | 0.00±0.00 | 94.52±30.00 |
| 0529_1452_E24 & Q24 | 12.42±0.17 | 48.9±10.0 | 13.22±1.25 | 83.74±5.34 | 311.01±333.39 | 5.18±1.83 | 0.00±0.00 | 49.59±20.03 |
| 0531_0840_E36 & Q36 | 14.11±0.23 | 66.8±10.7 | 12.76±1.04 | 84.50±5.34 | 229.12±311.63 | 6.04±1.83 | 0.00±0.00 | 48.71±22.54 |
| 0602_1013_E48 & Q48 | 15.05±0.29 | 40.3±12.8 | 17.47±2.79 | 65.02±15.11 | 323.76±333.33 | 4.51±1.58 | 0.00±0.00 | 75.92±35.63 |
| 0605_2109_E58.7 & Q58.7 | 16.29±0.27 | 52.1±20.3 | 15.14±1.56 | 96.52±5.48 | 206.22±249.01 | 4.77±2.01 | 0.02±0.07 | 279.69±76.42 |
| 0611_0950_E72 & Q72 | 17.73±0.35 | 33.3±16.4 | 16.49±1.86 | 80.13±9.81 | 300.44±325.28 | 3.48±1.18 | 0.00±0.00 | 261.55±39.87 |
| 0716_1840_Q8 | 18.62±0.15 | 96.22±4.16 | 17.25±0.45 | 71.70±1.35 | 511.70±161.85 | 6.98±0.57 | 0.00±0.00 | 173.72±174.69***** |
| 0728_2140_Q12 | 20.21±0.09 | 91.64±16.64 | 18.79±1.07 | 68.85±11.50 | 394.20±171.83 | 7.56±1.04 | 0.02±0.14 | 226.40±15.57 |
| 0806_1800_Q6 | 19.64±0.06 | 90.07±19.72 | 19.10±1.24 | 71.26±11.08 | 414.05±128.91 | 7.87±0.82 | 0.00±0.02 | 224.98±10.96 |
| 0807_1830_Q8 | 19.50±0.09 | 40.24±7.49 | 18.44±0.87 | 71.04±8.13 | 349.71±193.75 | 6.23±1.00 | 0.04±0.16 | 187.16±17.39 |
| 0808_1719_Q6 | 19.31±0.10 | 101.62±9.45 | 17.73±1.26 | 71.65±8.38 | 421.70±214.53 | 7.84±1.04 | 0.02±0.09 | 223.16±13.47 |
| 0809_1745_Q8 | 18.97±0.15 | 65.89±13.71 | 18.14±1.69 | 75.35±8.63 | 489.98±191.82 | 6.66±0.87 | 0.02±0.05 | 224.43±22.91 |
| 0812_2105_Q12 | 19.30±0.20 | 29.48±4.54 | 18.93±0.56 | 76.37±2.83 | 363.67±190.07 | 2.52±0.94 | 0.00±0.00 | 272.19±77.40 |
| 0818_1800_Q6 | 18.81±0.09 | 76.77±8.22 | 18.53±0.73 | 80.86±2.13 | 394.70±141.64 | 5.95±1.09 | 0.00±0.00 | 261.39±7.87 |
| 0901_1320_Q4 | 18.41±0.06 | 87.44±2.31 | 17.98±0.59 | 66.28±4.30 | 276.08±139.13 | 5.92±0.70 | 0.00±0.00 | 31.20±6.53 |
| 0902_1806_Q8.9 | 18.46±0.12 | 74.32±4.62 | 18.10±0.79 | 63.82±3.21 | 416.16±196.21 | 6.24±2.10 | 0.00±0.00 | 47.49±19.46 |
| 0903_1540_Q6 | 18.48±0.12 | 77.54±7.17 | 18.86±0.81 | 80.46±1.50 | 586.59±125.42 | 6.86±1.31 | 0.00±0.00 | 34.19±7.98 |
| 0915_1730_Q4 | 18.91±0.03 | 73.29±5.01 | 17.84±0.07 | 83.00±1.72 | 159.32±36.18 | 7.13±0.51 | 0.00±0.00 | 329.95±3.07 |

**Table S6.** Conditions of DNA extraction, full-length 16S rRNA gene amplification, and Nanopore sequencing for each aerosol sample.

- DNA extraction: Three-quarters of each sample filter was used.
- Blank filters: Total DNA yield and PCR-amplified full-length 16S rRNA gene yield from blank filters were below the detection limit; no visible PCR product bands were observed. Consequently, blank filters were excluded from sequencing.
- Filtering: ‘Filtered reads’ refer to the number of sequences remaining after removal of reads unassigned at the phylum level and those classified as chloroplasts or mitochondria; ‘Final reads’ refer to the number of sequences remaining after further excluding, from the ‘Filtered reads’, reads belonging to OTUs with fewer than 5 reads to reduce noise.
- LOD, limit of detection; NA, not available. “YES” in the column “Passed sequencing” indicates samples whose rarefaction curves reached a plateau (**Figure S2**).

| Sample ID | DNA yield (ng/m^3^ air) | PCR replicates | PCR bands | Purified PCR for sequencing (ng) | Sequencing batch | Barcode | Initial reads | Filtered reads | Final reads | Passed sequencing |
| --- | --- | --- | --- | --- | --- | --- | --- | --- | --- | --- |
| 0525_1222_E1 | 1.469 | 1 | Yes | 6 | 1 | 9 | 2,461 | 2,100 | 2,083 | No |
| 0525_1222_Q1 | Below LOD | 1 | No | 6 | 1 | 2 | 84 | 47 | 43 | No |
| 0525_1530_E2 | 0.378 | 1 | Yes | 3.384 | 1 | 3 | 90 | 53 | 43 | No |
| 0525_1530_Q2 | Below LOD | 1 | Yes | 3.42 | 1 | 4 | 658 | 499 | 491 | No |
| 0525_1925_E3 | Below LOD | 1 | No | 6 | 1 | 5 | NA | NA | NA | No |
| 0525_1925_Q3 | Below LOD | 1 | Yes | 6 | 1 | 6 | 1,988 | 1,882 | 1,862 | No |
| 0526_1325_E4 | Below LOD | 1 | Yes | 6 | 1 | 7 | 11,444 | 10,844 | 10,840 | Yes |
| 0526_1325_Q4 | Below LOD | 1 | Yes | 6 | 1 | 8 | 495 | 468 | 459 | No |
| 0526_2020_E6 | Below LOD | 1 | Yes | 6 | 1 | 1 | 59,301 | 45,234 | 45,208 | Yes |
| 0526_2020_Q6 | Below LOD | 1 | Yes | 6 | 1 | 10 | 18,972 | 16,113 | 16,080 | Yes |
| 0527_1601_E8 | Below LOD | 1 | Yes | 6 | 1 | 11 | 10,584 | 9,636 | 9,628 | Yes |
| 0527_1601_Q8 | Below LOD | 1 | Yes | 6 | 1 | 12 | 25,384 | 23,271 | 23,265 | Yes |
| 0528_0800_E12 | Below LOD | 1 | Yes | 6 | 1 | 13 | 12,472 | 12,459 | 12,455 | Yes |
| 0528_0800_Q12 | Below LOD | 1 | Yes | 6 | 1 | 14 | 11,243 | 11,132 | 11,126 | Yes |
| 0529_1452_E24 | 0.043 | 1 | Yes | 6 | 1 | 15 | 10,481 | 9,332 | 9,298 | Yes |
| 0529_1452_Q24 | 0.159 | 1 | Yes | 6 | 1 | 16 | 4,503 | 3,991 | 3,972 | Yes |
| 0531_0840_E36 | 0.053 | 1 | Yes | 25.8 | 1 | 17 | 100,902 | 93,966 | 93,945 | Yes |
| 0531_0840_Q36 | 0.097 | 1 | Yes | 31.4 | 1 | 18 | 60,621 | 54,059 | 53,997 | Yes |
| 0602_1013_E48 | 0.220 | 1 | No | 6 | 1 | 19 | 2 | 2 | 0 | No |
| 0602_1013_Q48 | 0.306 | 1 | No | 6 | 1 | 20 | NA | NA | NA | No |
| 0605_2109_E58.7 | 0.042 | 1 | Yes | 6 | 1 | 21 | 12,353 | 11,061 | 11,047 | Yes |
| 0605_2109_Q58.7 | 0.075 | 1 | Yes | 6 | 1 | 22 | 1,607 | 1,330 | 1,330 | No |
| 0611_0950_E72 | NA | NA | NA | NA | NA | NA | NA | NA | NA | No |
| 0611_0950_Q72 | 0.077 | 1 | Yes | 31.6 | 1 | 23 | 72,332 | 65,153 | 65,117 | Yes |
| 0716_1840_Q8 | Below LOD | 3 | Yes | 6 | 3 | 18 | 5,237 | 4,258 | 4,250 | Yes |
| 0728_2140_Q12 | 0.497 | 3 | Yes | 6 | 3 | 15 | 7,464 | 6,190 | 6,178 | Yes |
| 0806_1800_Q6 | Below LOD | 3 | Yes | 6 | 3 | 9 | 5,350 | 4,885 | 4,877 | Yes |
| 0807_1830_Q8 | Below LOD | 3 | Yes | 6 | 3 | 17 | 9,720 | 9,436 | 9,416 | Yes |
| 0808_1719_Q6 | Below LOD | 3 | Yes | 6 | 3 | 19 | 4,738 | 4,067 | 4,045 | Yes |
| 0809_1745_Q8 | 0.322 | 3 | Yes | 6 | 3 | 20 | 5,880 | 4,816 | 4,809 | Yes |
| 0812_2105_Q12 | Below LOD | 1 | Yes | 6 | 5 | 23 | 5,882 | 5,743 | 5,732 | Yes |
| 0818_1800_Q6 | Below LOD | 3 | Yes | 6 | 3 | 22 | 4,471 | 4,035 | 4,018 | Yes |
| 0901_1320_Q4 | 0.170 | 1 | Yes | 6 | 4 | 15 | 19,991 | 15,793 | 15,757 | Yes |
| 0902_1806_Q8.9 | 0.141 | 1 | Yes | 6 | 4 | 17 | 15,157 | 12,130 | 12,104 | Yes |
| 0903_1540_Q6 | 0.838 | 1 | Yes | 6 | 4 | 19 | 17,778 | 14,054 | 14,001 | Yes |
| 0915_1730_Q4 | 0.264 | 1 | Yes | 6 | 4 | 23 | 13,067 | 11,729 | 11,722 | Yes |

**Table S7.** Grouping of habitats in the ProkAtlas analysis.

- ProkAtlas provides proportional values of 107 detailed habitats. For interpretation, the 107 habitats were grouped into 9 broad habitat categories: ocean-related, freshwater-related, soil- and plant-related, human-related, animal-related, wastewater-related, industrial-related, extreme environments, and other habitats.

| Habitat (summarized) | Habitat (prokAtlas output) |
| --- | --- |
| ocean_related | marine, seawater, marine_sediment, salt_marsh, estuary, sponge, coral, sea_squirt, beach_sand |
| freshwater_related | freshwater, aquifer, freshwater_sediment, groundwater, lake_water, wetland, peat, rice_paddy |
| soil_and_plant_related | soil, rhizosphere, root, plant, endophyte, phyllosphere |
| human_related | human, human_gut, human_skin, human_eye, human_oral, human_lung, human_bile, human_reproductive_system, human_blood |
| animals_related | crab, annelid, crustacean, ant, chicken_gut, termite_gut, insect, insect_gut, bat, pig_gut, bovine_gut, mouse_gut, bird, oyster, rat_gut, fish_gut, mosquito, bovine, invertebrate |
| wastewater_related | wastewater, activated_sludge, biosolids, bioreactor, bioreactor_sludge, anaerobic_digester, biofilm, biofilter, sludge, mine_drainage |
| industrial_related | biogas_fermenter, activated_carbon, fermentation, food, food_fermentation, food_production, compost, microbial_fuel_cell, paper_pulp |
| extreme environments | hydrothermal_vent, salt_lake, glacier, permafrost, hypersaline_lake, fossil, hot_springs, oil_field, hydrocarbon, hypolithon |
| others | aquatic, sediment, terrestrial, urban, indoor, subsurface, surface, rock, rock_porewater, cave, gut, skin, oral, feces, symbiont, epibiont, parasite, algae, fungus, viral, lichen, moss, stromatolite, microbial_mat, landfill, mine_tailings, money |

**Table S8.** Water quality monitoring data for coastal waters at Ostend – Dunes and Sea (51°14′34′′N, 2°56′12′′E; ~953 m from the aerosol sampling site), collected by the Flemish Environment Agency and the Department of Care, during the aerosol sampling period of this study.

- Data were obtained from <https://kwaliteitzwemwater.be>.
- According to the European Bathing Water Directive (2006/7/EC), the quality levels of coastal water quality are:
  - Very good: intestinal *enterococci*/100 mL: ≤200 (control sample required), *E. coli*/100 mL: ≤500 (control sample required), swimming advice: No problems.
  - Acceptable: intestinal *enterococci*/100 mL: ≤400 (control sample required), *E. coli*/100 mL: ≤1,000 (control sample required), swimming advice: Swimming is not recommended for young children, the elderly and people with low resistance.
  - Bad: intestinal *enterococci*/100 mL: >400 (control sample required), *E. coli*/100 mL: >1,000 (control sample required), swimming advice: Swimming is not recommended for young children, the elderly and people with low resistance.

Reference: <https://kwaliteitzwemwater.be/nl/normen>.

| Date | Quality level | Intestinal *enterococci* | *E. coli* |
| --- | --- | --- | --- |
| 20210920 | Very good | 19 | 40 |
| 20210913 | Very good | 1 | 12 |
| **20210910** | **Bad** | **486** | **444** |
| 20210906 | Very good | 20 | 57 |
| 20210902 | Very good | 3 | 5 |
| 20210831 | Very good | 8 | 14 |
| 20210827 | Very good | 48 | 56 |
| 20210826 | Very good | 40 | 58 |
| **20210823** | **Acceptable** | 198 | **860** |
| 20210817 | Very good | 44 | 88 |
| 20210813 | Very good | 7 | 43 |
| 20210809 | Very good | 6 | 27 |
| 20210803 | Very good | 9 | 9 |
| 20210730 | Very good | 11 | 58 |
| 20210726 | Very good | 50 | 62 |
| 20210720 | Very good | 6 | 17 |
| 20210716 | Very good | 11 | 28 |
| 20210712 | Very good | 16 | 46 |
| 20210706 | Very good | 1 | 25 |
| 20210702 | Very good | 6 | 131 |
| 20210628 | Very good | 113 | 422 |
| 20210622 | Very good | 16 | 82 |
| 20210618 | Very good | 3 | 13 |
| 20210614 | Very good | 9 | 16 |
| 20210608 | Very good | 43 | 47 |
| 20210604 | Very good | 6 | 51 |
| 20210517 | Very good | 4 | 1 |


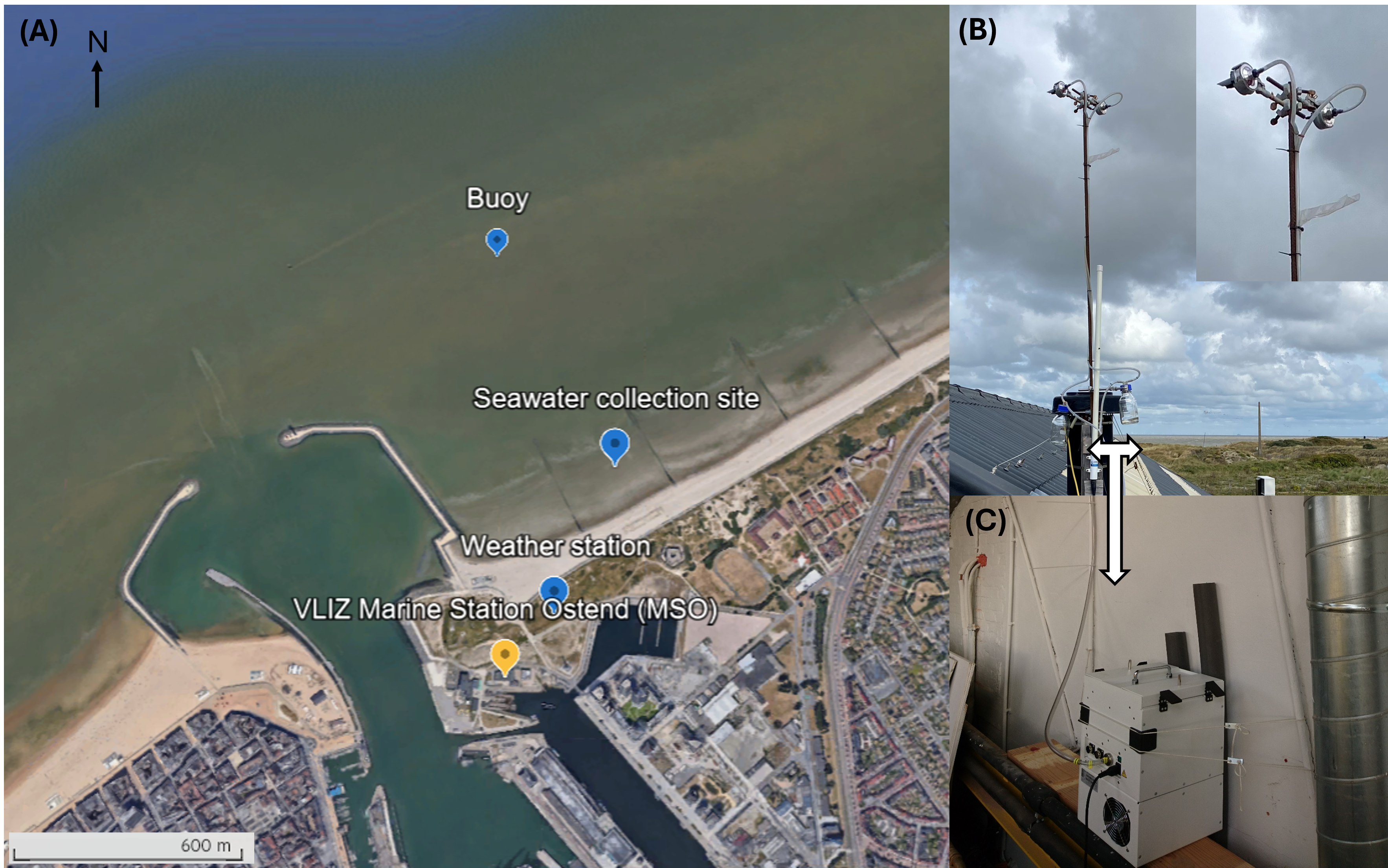


**Figure S1.** (A) Map showing the location of the Marine Station Ostend (MSO; 51°14′10′′N, 2°55′42′′E) for aerosol sampling and processing, as well as nearby environmental monitoring stations (Buoy: 51°14′48”N, 2°55′39″E; Weather station: 51°14′15”N, 2°55′48″E). The map also marks the seawater collection site (approximately 51°14'28''N, 2°55'56''E) reported by Li et al. (2024), whose seawater bacterial data collected weekly from March 2018 to March 2019 were used for comparison in this study. Sea wind directions at MSO range between ≥235° and ≤58°(Van Acker et al., 2021). (B,C) Aerosol sampling setup on the rooftop (10 m above ground level) of Warehouse 4 at MSO. The setup includes an AirCube air sampler positioned indoors near the roof (C), with its air intake connected to a tube that branched into two via a Y-joint. Each branch was connected to one of two side-by-side filter holders mounted on a 1-m-high iron pole secured to the rooftop (B). One holder contains a glass fiber filter (EPM2000), and the other a quartz fiber filter (QM-A).


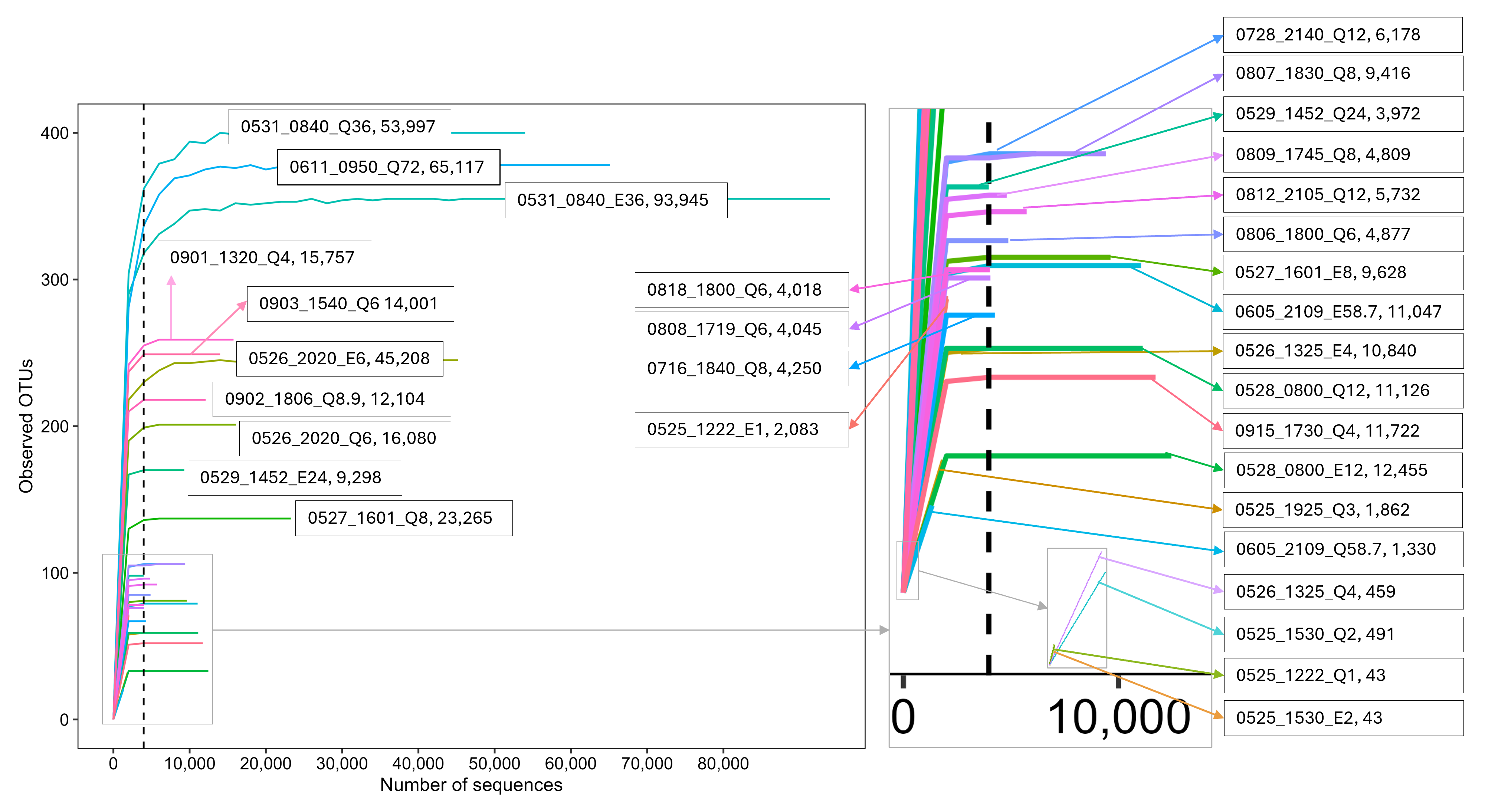


**Figure S2.** Rarefaction curves showing observed OTUs as a function of sequencing depth for each aerosol sample. The dotted line indicates the minimum sequencing depth (3,972 reads) at which the rarefaction curves of samples that passed sequencing reached a plateau.

**
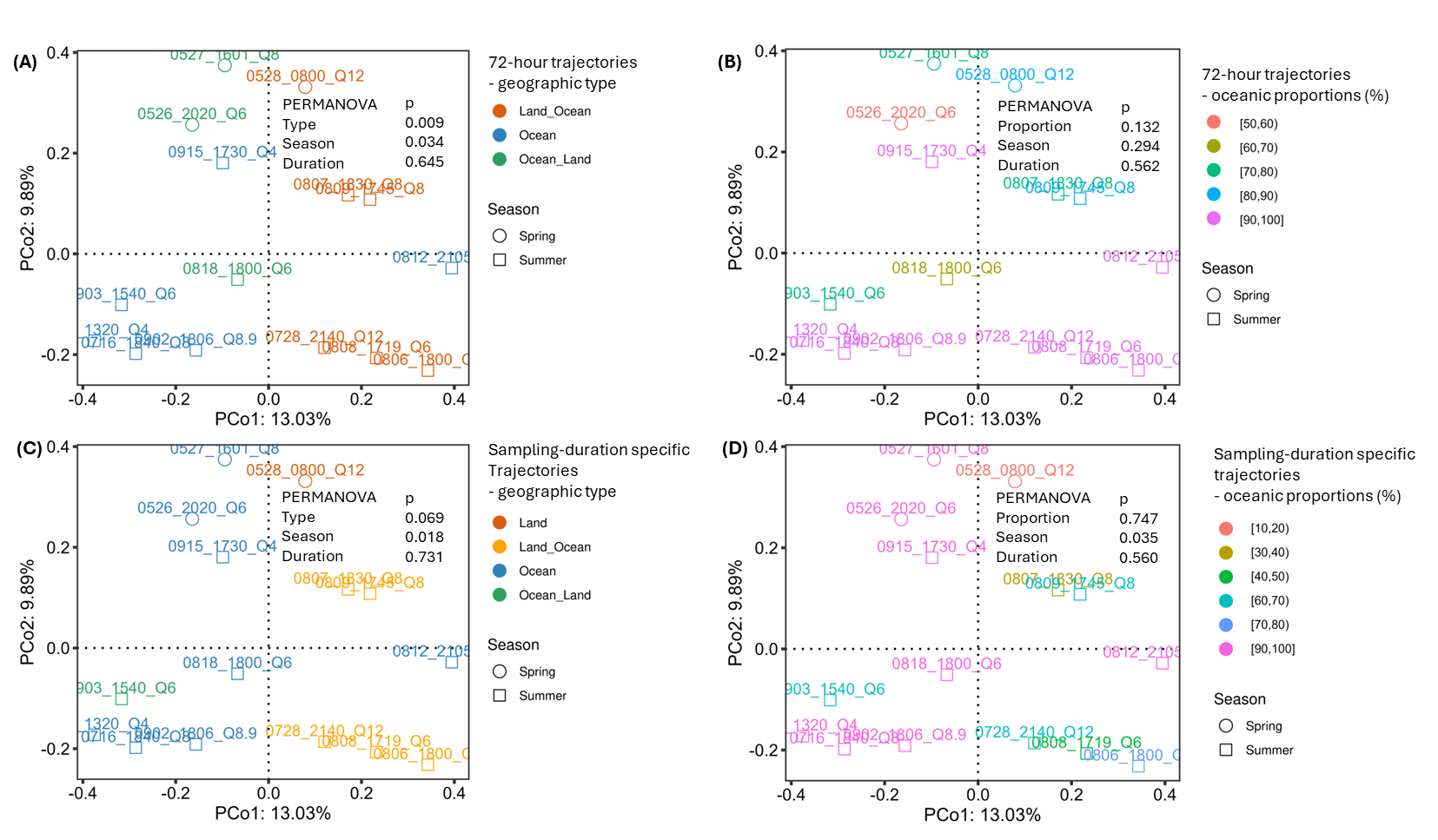
**

**Figure S3.** PCoA plots and PERMANOVA results showing the effects of geographic trajectory type and oceanic exposure proportion, derived from 72 h and sampling-duration-specific back trajectories, on airborne bacterial community composition, with season and sampling duration included as additional factors.


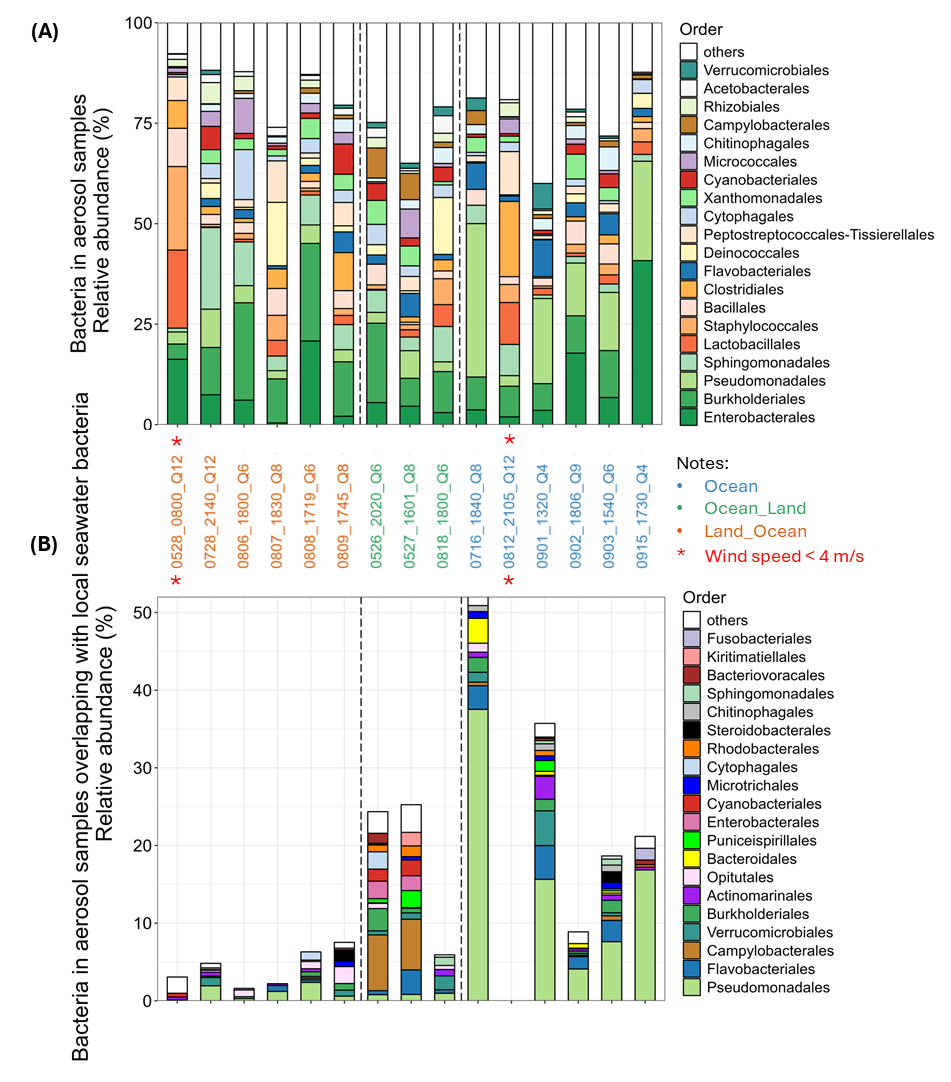


**Figure S4.** (A) Top 20 orders of bacteria in aerosol samples. (B) Top 20 orders of bacteria in aerosol samples overlapping with local seawater bacteria. Samples are grouped as Ocean, Ocean_Land, and Land_Ocean based on the geographic types derived from 72 h air mass back trajectories.

**
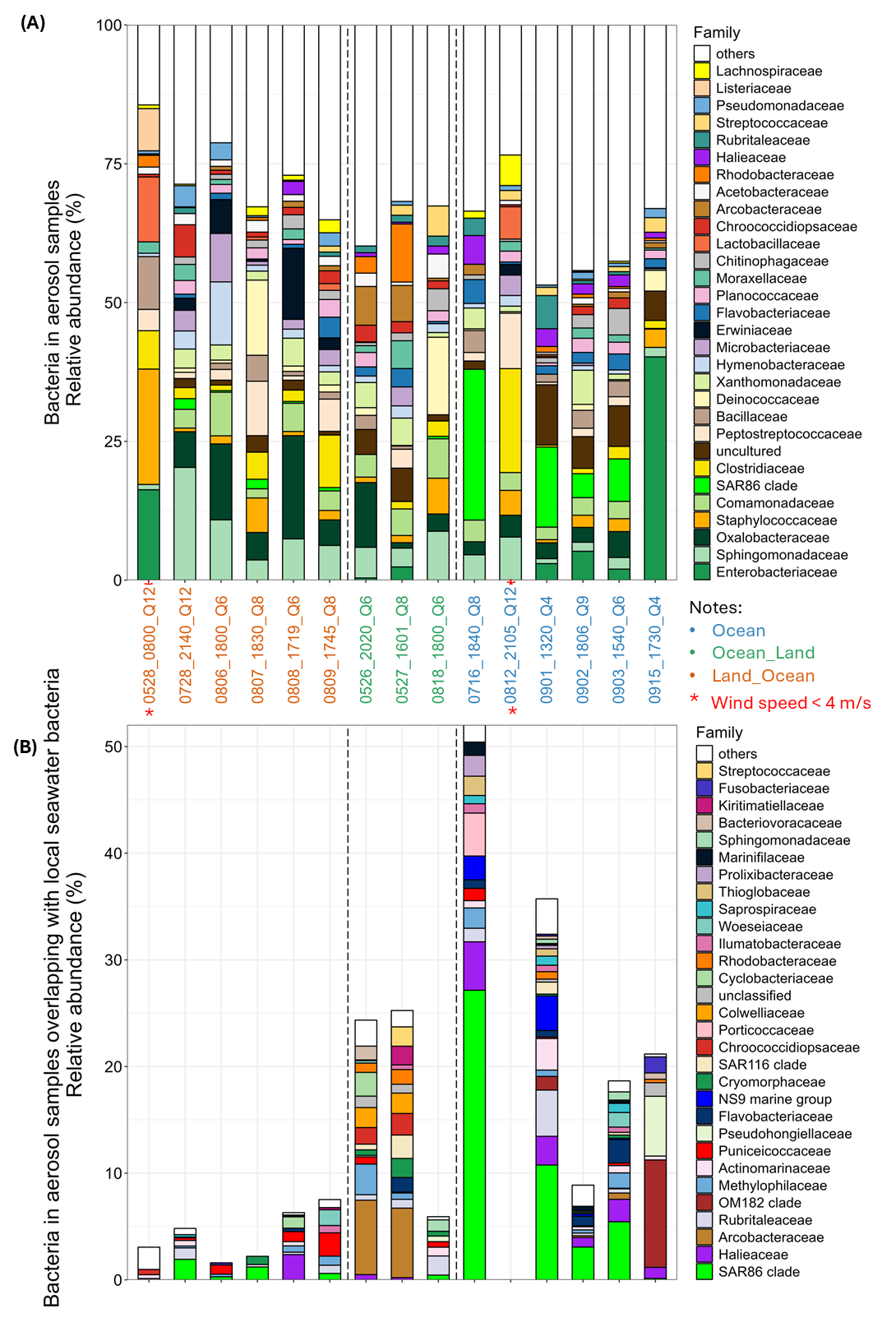
**

**Figure S5.** (A) Top 30 families of bacteria in aerosol samples. (B) Top 30 families of bacteria in aerosol samples overlapping with local seawater bacteria. Samples are grouped as Ocean, Ocean_Land, and Land_Ocean based on the geographic types derived from 72 h air mass back trajectories.


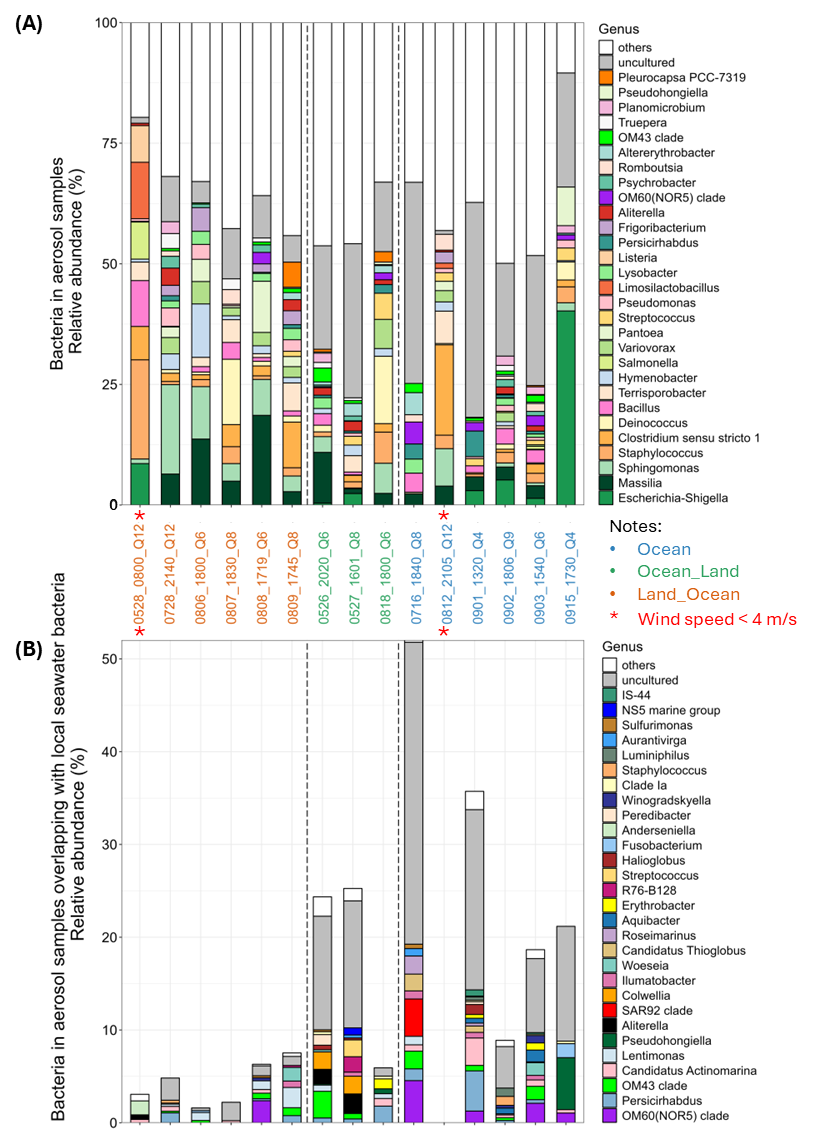


**Figure S6.** (A) Top 30 genera in aerosol samples. (B) Top 30 genera of bacteria in aerosol samples overlapping with local seawater bacteria. Samples are grouped as Ocean, Ocean_Land, and Land_Ocean based on the geographic types derived from 72 h air mass back trajectories.


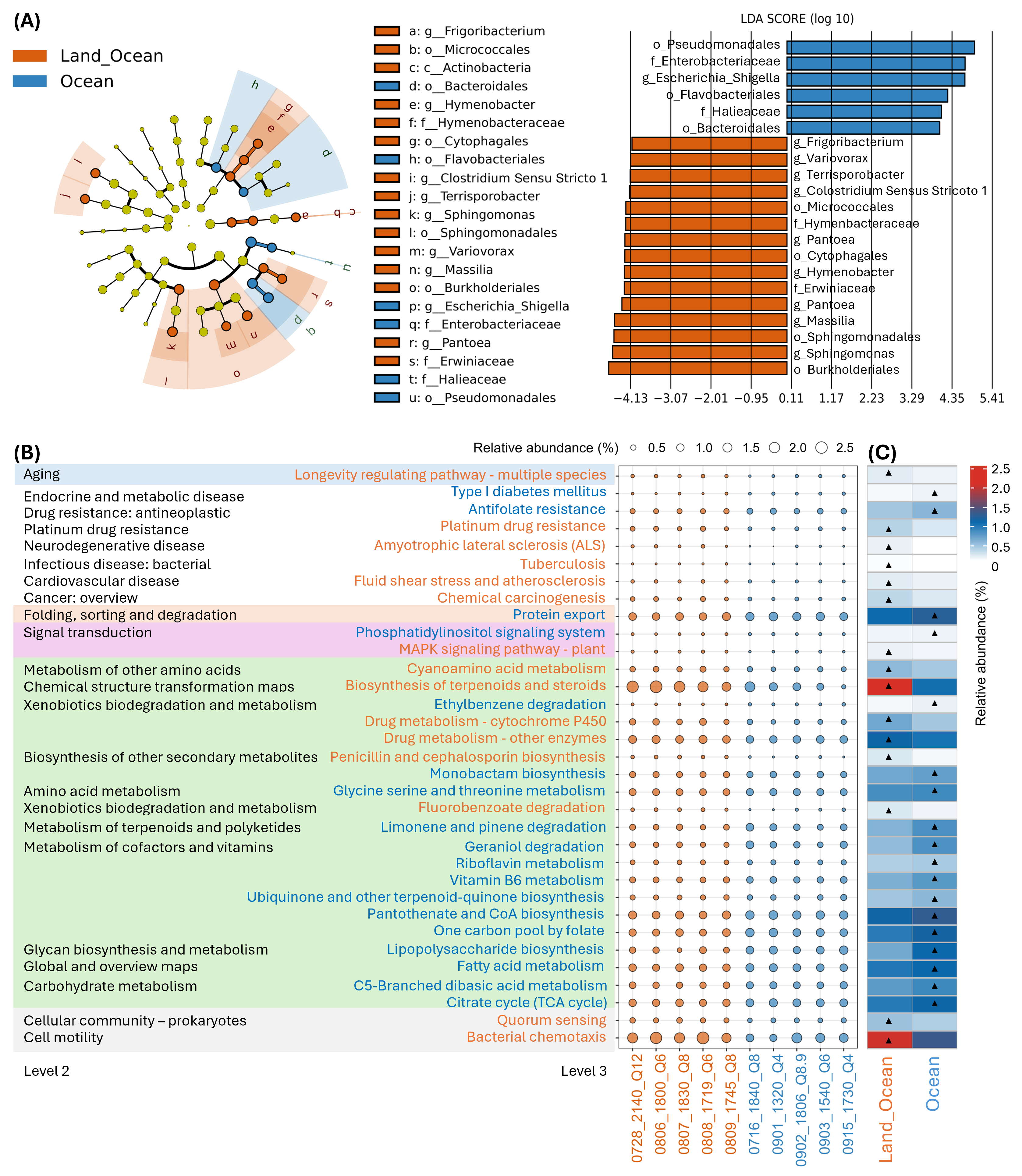


**Figure S7.** (A) Linear discriminant analysis (LDA) effect size (LEfSe) identifying bacterial taxa from phylum to genus that differed between the Ocean and Land_Ocean groups, using an LDA score cutoff of 4 and a significance threshold of *p* < 0.05. (B,C) Differences in PICRUSt2-inferred bacterial metabolic functions (level 3; relative abundance >0.1%) between the Ocean and Land_Ocean groups. Significances were tested using the Wilcoxon rank-sum test (*p* < 0.05). Panel (B) shows significantly different functions and their corresponding level-2 categories across individual samples, whereas panel (C) summarizes the average relative abundance of each function in the two groups. Triangles in panel (C) indicate the group in which each function is significantly more abundant.


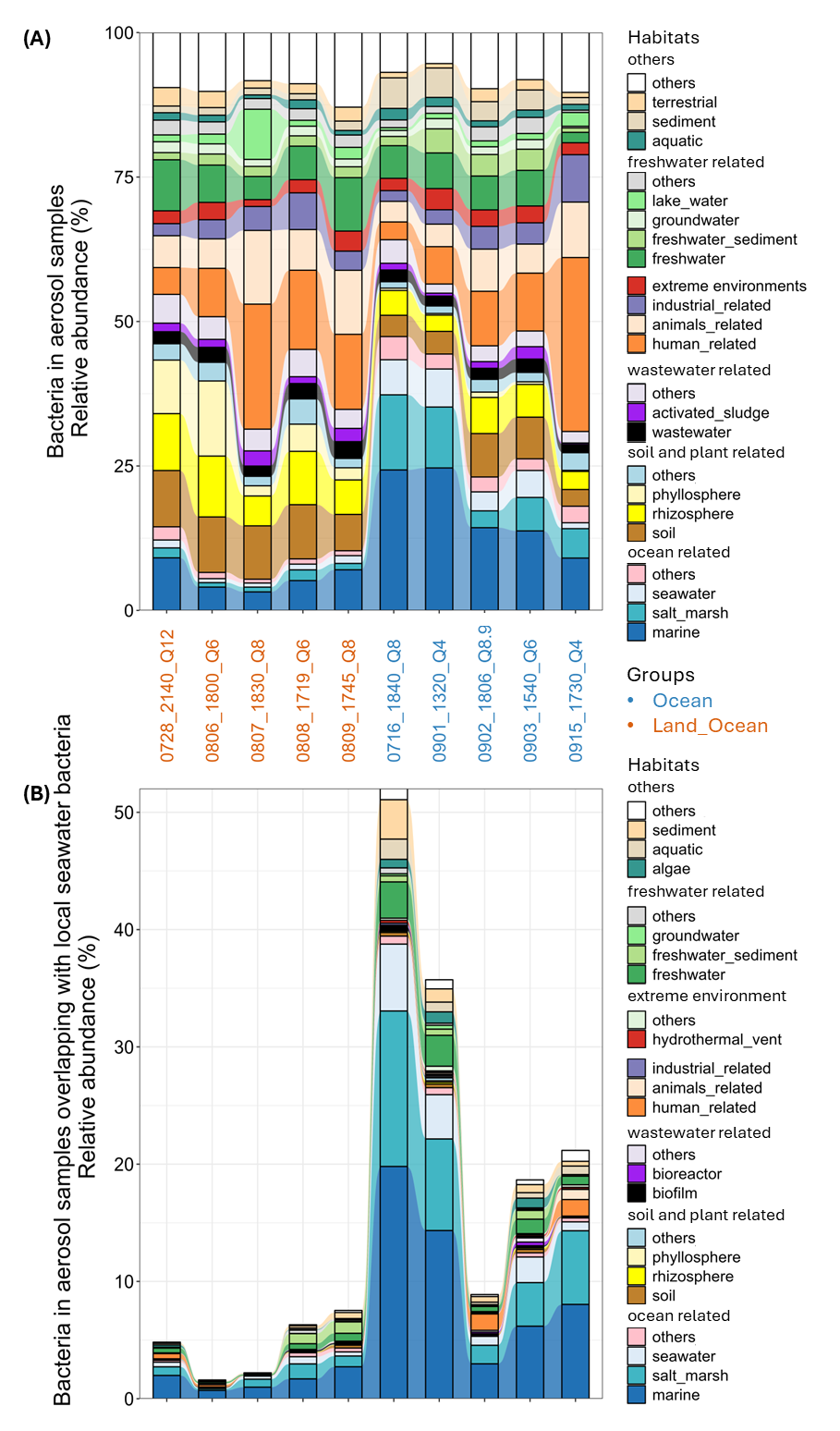


**Figure S8.** Preferential habitats inferred by ProkAtlas for (A) bacteria in aerosol samples and (B) bacteria in aerosol samples overlapping with local seawater bacteria. ProkAtlas predicts potential habitat affiliations across various environmental settings.

**References**

Abdool-Ghany, A.A., Klaus, J.S., Sosa Villegas, L.E., D’Alessio, T., Gidley, M.L., Sinigalliano, C.D., Gaston, C., Solo-Gabriele, H.M., 2023. Microbial communities in the water surface microlayer and associations with microbes in aerosols, beach sand, and bulk water. FEMS Microbiol. Ecol. 99, p.fiad039. https://doi.org/10.1093/femsec/fiad039

Archer, S.D.J.J., Lee, K.C., Caruso, T., King-Miaow, K., Harvey, M., Huang, D., Wainwright, B.J., Pointing, S.B., 2020. Air mass source determines airborne microbial diversity at the ocean-atmosphere interface of the Great Barrier Reef marine ecosystem. ISME J. 14, 871–876. https://doi.org/10.1038/s41396-019-0555-0

Cao, Y., Yu, X., Ju, F., Zhan, H., Jiang, B., Kang, H., Xie, Z., 2021. Airborne bacterial community diversity, source and function along the Antarctic Coast. Sci. Total Environ. 765, 142700. https://doi.org/10.1016/j.scitotenv.2020.142700

Graham, K.E., Prussin, A.J., Marr, L.C., Sassoubre, L.M., Boehm, A.B., Prussin II, A.J., Marr, L.C., Sassoubre, L.M., Boehm, A.B., 2018. Microbial community structure of sea spray aerosols at three California beaches. FEMS Microbiol. Ecol. 94, p.fiy005. https://doi.org/10.1093/femsec/fiy005

He, Q., Wang, Z., Liu, H., Xu, P., Duan, R., Xu, C., Chen, J., Wei, M., 2024. Impact assessment of terrestrial and marine air-mass on the constituents and intermixing of bioaerosols over coastal atmosphere. Egusph. [preprint] 5.

Jang, J., Park, J., Hwang, C.Y., Gim, Y., Park, K.T., Yoon, Y.J., Seo, M., Lee, B.Y., 2024. Selective transmission of airborne bacterial communities from the ocean to the atmosphere over the Northern Pacific Ocean. Sci. Total Environ. 957. https://doi.org/10.1016/j.scitotenv.2024.177462

Lang-Yona, N., Flores, J.M., Haviv, R., Alberti, A., Poulain, J., Belser, C., Trainic, M., Gat, D., Ruscheweyh, H.J., Wincker, P., Sunagawa, S., Rudich, Y., Koren, I., Vardi, A., 2022. Terrestrial and marine influence on atmospheric bacterial diversity over the north Atlantic and Pacific Oceans. Commun. Earth Environ. 3, 121. https://doi.org/10.1038/s43247-022-00441-6

Li, Y., Hablützel, P.I., Acker, E. Van, Janssen, C.R., Asselman, J., De Rijcke, M., 2024. Seasonal dynamics of bacterial community structure and function in the surf zone seawater of a recreational beach in Ostend , Belgium. Environ. Microbiol. Rep. 16, e70031. https://doi.org/10.1111/1758-2229.70031

Ma, M., Zhang, B., Chen, Y., Feng, W., Mi, T., Qi, J., Li, W., Yu, Z., Zhen, Y., 2021. Characterization of bacterial communities during persistent fog and haze events in the Qingdao coastal region. Front. Environ. Sci. Eng. 15. https://doi.org/10.1007/s11783-020-1334-x

Ma, M., Zhen, Y., Mi, T., 2019. Characterization of bacterial communities in bioaerosols over Northern Chinese Marginal Seas and the Northwestern Pacific Ocean in spring. J. Appl. Meteorol. Climatol. 58, 903–917. https://doi.org/10.1175/JAMC-D-18-0142.1

Mescioglu, E., Rahav, E., Belkin, N., Xian, P., Eizenga, J.M., Vichik, A., Herut, B., Paytan, A., 2019. Aerosol microbiome over the Mediterranean Sea diversity and abundance. Atmosphere (Basel). 10. https://doi.org/10.3390/atmos10080440

Palladino, G., Morozzi, P., Biagi, E., Brattich, E., Turroni, S., Rampelli, S., Tositti, L., Candela, M., 2021. Particulate matter emission sources and meteorological parameters combine to shape the airborne bacteria communities in the Ligurian coast, Italy. Sci. Rep. 11, 175. https://doi.org/10.1038/s41598-020-80642-1

Pendergraft, M.A., Grimes, D.J., Giddings, S.N., Feddersen, F., Beall, C.M., Lee, C., Santander V, M., Prather, K.A., 2021. Airborne transmission pathway for coastal water pollution. PeerJ 9. https://doi.org/10.7717/peerj.11358

Seifried, J.S., Wichels, A., Gerdts, G., 2015. Spatial distribution of marine airborne bacterial communities. Microbiologyopen 4, 475–490. https://doi.org/10.1002/mbo3.253

Tastassa, A.C., Dubowski, Y., Argaman Meirovich, O., Kuzmenkov, I., Lang-Yona, N., 2025. Selective ocean–atmosphere bacterial flux through the Pacific sea surface microlayer. ACS ES&T Air 2, 837–846. https://doi.org/10.1021/acsestair.4c00302

Tignat-Perrier, R., Dommergue, A., Thollot, A., Keuschnig, C., Magand, O., Vogel, T.M., Larose, C., 2019. Global airborne microbial communities controlled by surrounding landscapes and wind conditions. Sci. Rep. 9, 14441. https://doi.org/10.1038/s41598-019-51073-4

Uetake, J., Hill, T.C.J., Moore, K.A., DeMott, P.J., Protat, A., Kreidenweis, S.M., 2020. Airborne bacteria confirm the pristine nature of the Southern Ocean boundary layer. Proc. Natl. Acad. Sci. 202000134. https://doi.org/10.1073/pnas.2000134117

Uetake, J., Tobo, Y., Uji, Y., Hill, T.C.J.J., DeMott, P.J., Kreidenweis, S.M., Misumi, R., 2019. Seasonal changes of airborne bacterial communities over Tokyo and influence of local meteorology. Front. Microbiol. 10. https://doi.org/10.3389/fmicb.2019.01572

Van Acker, E., De Rijcke, M., Liu, Z., Asselman, J., De Schamphelaere, K.A.C., Vanhaecke, L., Janssen, C.R., 2021. Sea spray aerosols contain the major component of human lung surfactant. Environ. Sci. Technol. 55, 15989–16000. https://doi.org/10.1021/acs.est.1c04075

Wang, F., Chen, Y., Zhou, S., Li, H., Wan, C., Yan, K., Zhang, H., Xu, Z., 2024. Aerosol sources and transport paths co-control the atmospheric bacterial diversity over the coastal East China Sea. Mar. Pollut. Bull. 205, 116589. https://doi.org/10.1016/j.marpolbul.2024.116589

Wei, M., Liu, H., Chen, J., Xu, C., Li, J., Xu, P., Sun, Z., 2020. Effects of aerosol pollution on PM2.5-associated bacteria in typical inland and coastal cities of northern China during the winter heating season. Environ. Pollut. 262, 114188. https://doi.org/10.1016/j.envpol.2020.114188

Xia, X., Wang, J., Ji, J., Zhang, J., Chen, L., Zhang, R., 2015. Bacterial communities in marine aerosols revealed by 454 pyrosequencing of the 16S rRNA gene. J. Atmos. Sci. 72, 2997–3008. https://doi.org/10.1175/JAS-D-15-0008.1

Zhang, B., Zhen, Y., Mi, T., Qi, J., Yuan, G., 2023. Characterization of bacterial communities in aerosols over Northern Chinese Marginal Seas and the Northwestern Pacific Ocean in autumn. J. Ocean Univ. China 22, 136–150. https://doi.org/10.1007/s11802-023-5243-z
